# Meiosis-Specific Cohesin Component, *Stag3* Is Essential for Maintaining Centromere Chromatid Cohesion, and Required for DNA Repair and Synapsis between Homologous Chromosomes

**DOI:** 10.1371/journal.pgen.1004413

**Published:** 2014-07-03

**Authors:** Jessica Hopkins, Grace Hwang, Justin Jacob, Nicklas Sapp, Rick Bedigian, Kazuhiro Oka, Paul Overbeek, Steve Murray, Philip W. Jordan

**Affiliations:** 1 Department of Biochemistry and Molecular Biology, Johns Hopkins University Bloomberg School of Public Health, Baltimore, Maryland, United States of America; 2 The Jackson Laboratory, Bar Harbor, Maine, United States of America; 3 Department of Molecular and Cellular Biology, Baylor College of Medicine, Houston, Texas, United States of America, Department of Molecular and Human Genetics, Baylor College of Medicine, Houston, Texas, United States of America; University of California Davis, United States of America

## Abstract

Cohesins are important for chromosome structure and chromosome segregation during mitosis and meiosis. Cohesins are composed of two structural maintenance of chromosomes (SMC1-SMC3) proteins that form a V-shaped heterodimer structure, which is bridged by a α-kleisin protein and a stromal antigen (STAG) protein. Previous studies in mouse have shown that there is one SMC1 protein (SMC1β), two α-kleisins (RAD21L and REC8) and one STAG protein (STAG3) that are meiosis-specific. During meiosis, homologous chromosomes must recombine with one another in the context of a tripartite structure known as the synaptonemal complex (SC). From interaction studies, it has been shown that there are at least four meiosis-specific forms of cohesin, which together with the mitotic cohesin complex, are lateral components of the SC. STAG3 is the only meiosis-specific subunit that is represented within all four meiosis-specific cohesin complexes. In *Stag3* mutant germ cells, the protein level of other meiosis-specific cohesin subunits (SMC1β, RAD21L and REC8) is reduced, and their localization to chromosome axes is disrupted. In contrast, the mitotic cohesin complex remains intact and localizes robustly to the meiotic chromosome axes. The instability of meiosis-specific cohesins observed in *Stag3* mutants results in aberrant DNA repair processes, and disruption of synapsis between homologous chromosomes. Furthermore, mutation of *Stag3* results in perturbation of pericentromeric heterochromatin clustering, and disruption of centromere cohesion between sister chromatids during meiotic prophase. These defects result in early prophase I arrest and apoptosis in both male and female germ cells. The meiotic defects observed in *Stag3* mutants are more severe when compared to single mutants for *Smc1β*, *Rec8* and *Rad21l*, however they are not as severe as the *Rec8*, *Rad21l* double mutants. Taken together, our study demonstrates that STAG3 is required for the stability of all meiosis-specific cohesin complexes. Furthermore, our data suggests that STAG3 is required for structural changes of chromosomes that mediate chromosome pairing and synapsis, DNA repair and progression of meiosis.

## Author Summary

Meiosis is a specialized cell division required for the formation of gametes (sperm and egg). Early in meiosis, the chromosome pairs that we inherit from our mother and father become linked and genetic material is exchanged. This is a remarkable process as every gamete that we make is unique, and the unison between a sperm and egg will create a new individual that harbors novel combinations of characteristics from each parents' family tree. Linkage and genetic exchange between chromosomes is facilitated by a linear protein scaffold structure. A group of protein complexes known as cohesins are a key component of the protein scaffold. To date, there are 4 meiosis-specific cohesin complexes identified. Only one cohesin component known as STAG3 is represented in all meiosis-specific cohesins. We mutated the gene that encodes for STAG3 in mouse and discovered that it results in meiotic failure and absence of gametes. From careful analysis we have determined that STAG3 is required for the stability of meiosis-specific cohesins, which ensure that chromosomes are paired and genetic material is exchanged. Our findings imply that abnormalities in human STAG3 will give rise to chromosome defects, infertility and gonad atrophy.

## Introduction

During mitosis, chromosomes are replicated and the resulting sister chromatids are segregated, generating two genetically identical daughter cells. Meiosis, on the other hand, is a specialized cell division that involves chromosome replication and two rounds of chromosome segregation (meiosis I and II), resulting in the formation of up to four haploid gametes. Meiosis I differs from mitosis because homologous chromosomes segregate, whereas sister chromatids remain associated until meiosis II. In order to ensure successful chromosome segregation during meiosis I, three coordinated events occur during prophase I, namely homologous chromosome pairing, synapsis, and recombination [1].

In mitotic cells, a structural maintenance of chromosomes (SMC) complex known as cohesin is required to hold sister chromatids together prior to the metaphase to anaphase I transition. The mammalian mitotic cohesin complex is composed of a heterodimer between SMC1α and SMC3 that form a V-shaped structure that is bridged by an α-kleisin known as RAD21 (Radiation Sensitive 21) and a stromal antigen protein (STAG1 or STAG2) [2]. Meiosis-specific cohesin subunits have been characterized for most model organisms, and are required for the unique events that occur during prophase I. In mammals there is a meiosis-specific SMC1 subunit (SMC1β), two additional α-kleisins (RAD21L and REC8) and another stromal antigen protein (STAG3) [3]–[6]. Based on interaction studies there are at least five species of cohesin complex associated with chromosomes during meiosis, including the mitotic cohesin (SMC1α-SMC3 bridged by STAG1 or 2 and RAD21), meiosis-specific SMC1β-containing cohesins (SMC1β-SMC3 bridged by STAG3 and either RAD21, REC8 or RAD21L), and meiosis-specific SMC1α-containing cohesins (SMC1α-SMC3 bridged by STAG3 and RAD21L or possibly REC8). From these interaction data, STAG3 is the only subunit that it is present in all meiosis-specific cohesin complexes [3], [7], [8].

Mitotic and meiosis-specific cohesin components first localize to chromatin during pre-meiotic S phase (also known at the pre-leptotene stage [2], [3], [8]–[12]. During pre-leptotene, telomeres become anchored to the nuclear envelope and rapid chromosome movements that facilitate initial pairing of homologous chromosomes are observed [13], [14]. Meiosis-specific cohesins localize to the telomeres at this stage and are required for stable telomere anchoring to the nuclear periphery [15]–[17]. All mouse chromosomes are telocentric and STAG3, REC8 and RAD21L cohesins also localize to the heterochromatin rich pericentromeric clusters (“chromocenters”) that form during pre-leptotene and are thought to be required for chromosome pairing [3], [15]. Another SMC protein complex known as SMC5/6 was recently shown to localize to the chromocenters at this stage of meiotic progression [18], [19]. In mitotic cells, it has been shown that chromocenters play a critical role in centromere function, inhibition of DNA recombination and chromosome segregation [20], [21]. When mitotic cells progress through prophase, pericentromeric regions of each chromosome dissociate from one another [20]. In contrast, chromocenters remain prevalent throughout prophase I of meiosis and these clusters may be required for chromosome pairing and inhibition of aberrant DNA recombination events at highly repetitive sequences [18], [19].

During the leptotene sub-stage of prophase I the meiosis-specific topoisomerase II-like enzyme, SPO11 introduces DSBs. These DSBs stimulate a DNA damage response (DDR) signaling cascade directed by ataxia telangiectasia mutated (ATM) and Rad3-related (ATR) kinases. ATM and ATR phosphorylate histone H2AFX (γH2AX) [22], [23] and recruit other DDR proteins including the ATR interacting protein (ATRIP) [24]. Concurrently, all cohesin complexes together with HORMA (Hop1-Rev7-Mad2) domain containing proteins HORMAD1 and 2 and the synaptonemal complex (SC) proteins SYCP2 and SYCP3 form axial elements between sister chromatids [25]–[27]. DSB repair is initiated at the zygotene stage where DNA pairing and strand-exchange proteins RAD51 and DMC1 (disrupted meiotic cDNA) initiate inter-homolog recombination. The inter-homolog interactions are coupled with the formation of the SC, whereby axial elements become lateral elements of the SC, and central region proteins including SYCP1 and testis expressed protein 12 (TEX12) link the homologues together [28]. During SC formation HORMAD1 and 2 disassemble from regions that have synapsed [27]. At the pachytene stage, homologous chromosomes are fully synapsed, and DSB repair is complete, resulting in the formation of non-crossover and crossover events. During spermatogenesis, the largely non-homologous X-Y chromosomes synapse at the pseudo-autosomal region (PAR) and are transcriptionally silenced to form the X-Y body [29], [30]. SC disassembly occurs during the diplotene stage, when central region proteins only remain at sites of crossovers and chromosome ends, whereas lateral element components including cohesin remain associated along the length of the chromosomes [3]–[6], [31]. At the final sub-stage of prophase I, diakineses, lateral element proteins such as SYCP3 and cohesin components SMC1β, RAD21, REC8, STAG3 and RAD21L become more punctate on chromosome arms and more prominent at the centromeres [3]–[6]. However, there is currently an inconsistency in localization data reported for cohesin component RAD21L as it has also been shown to be removed from the chromosome arms either at mid-to late pachytene stage [8], [32] or by diakinesis [33].

Homozygous mouse mutants for meiosis-specific cohesin subunits *Smc1β*, *Rec8* and *Rad21L* have been characterized in both male and female mice. The aberrant meiotic phenotypes observed for each mutation were not identical. Mutation of *Smc1β* causes a mid-pachytene arrest in primary spermatocytes with shortened axial elements and failure to form crossovers [34] Female *Smc1β* mouse mutants on the other hand are fertile, but show correlation between increased incidence of non-disjunction and age, suggesting that there is a cohesin dependent mechanism for stabilizing sites of crossovers and centromeric cohesion [35]. Male mutants for Rad21l have a morphologically different zygotene-like arrest, exhibiting incomplete synapsis between homologues, a degree of synapsis between non-homologues and the absence of crossovers [16]. *Rad21l* female mutants are fertile, but they have premature ovarian failure which is linked to a defect in synapsis but not maintenance of chiasmata [16]. Male and female mouse mutants for *Rec8* result in a meiotic arrest characterized by an aberrant zygotene-like stage with synapsed sister chromatids and the absence of crossovers [36], [37]. *Rec8*, *Rad21l* double mutants result in a leptotene-like arrest and immunofluorescence observations suggest that only the mitotic cohesin localizes to the axial elements [12]. Localization of STAG3 to chromosome axes is observed in *Smc1β*, *Rec8* and *Rad21L* mutants, whereas a chromatin bound STAG3 signal was absent in the *Rec8*, *Rad21l* double mutants [12], [16], [34]–[37]. STAG3 is unique, as it is a component of all meiosis-specific cohesin complexes [3], [7], [8]. It is of great interest to assess how mutation of *Stag3* effects meiotic progression, in comparison to the other cohesin mutants previously characterized.

We used two independently created null mutations for *Stag3* and determined that STAG3 is required for clustering of pericentromeric heterochromatin, maintenance of centromere cohesion between sister chromatids, synapsis between homologues and repair of SPO11-induced DSBs. We show that STAG3 is required for normal axial localization and stability of meiosis-specific cohesin subunits SMC1β, REC8 and RAD21L. Mutation of *Stag3* results in a zygotene-like stage arrest, which is less severe than that reported for the *Rec8*, *Rad21l* double mutants. We hypothesize that localization of REC8 and RAD21L cohesins to chromosome axes are stabilized by STAG3.

## Results

### *Stag3* mutation results in sterility in male and female mice

**Figure 1:**
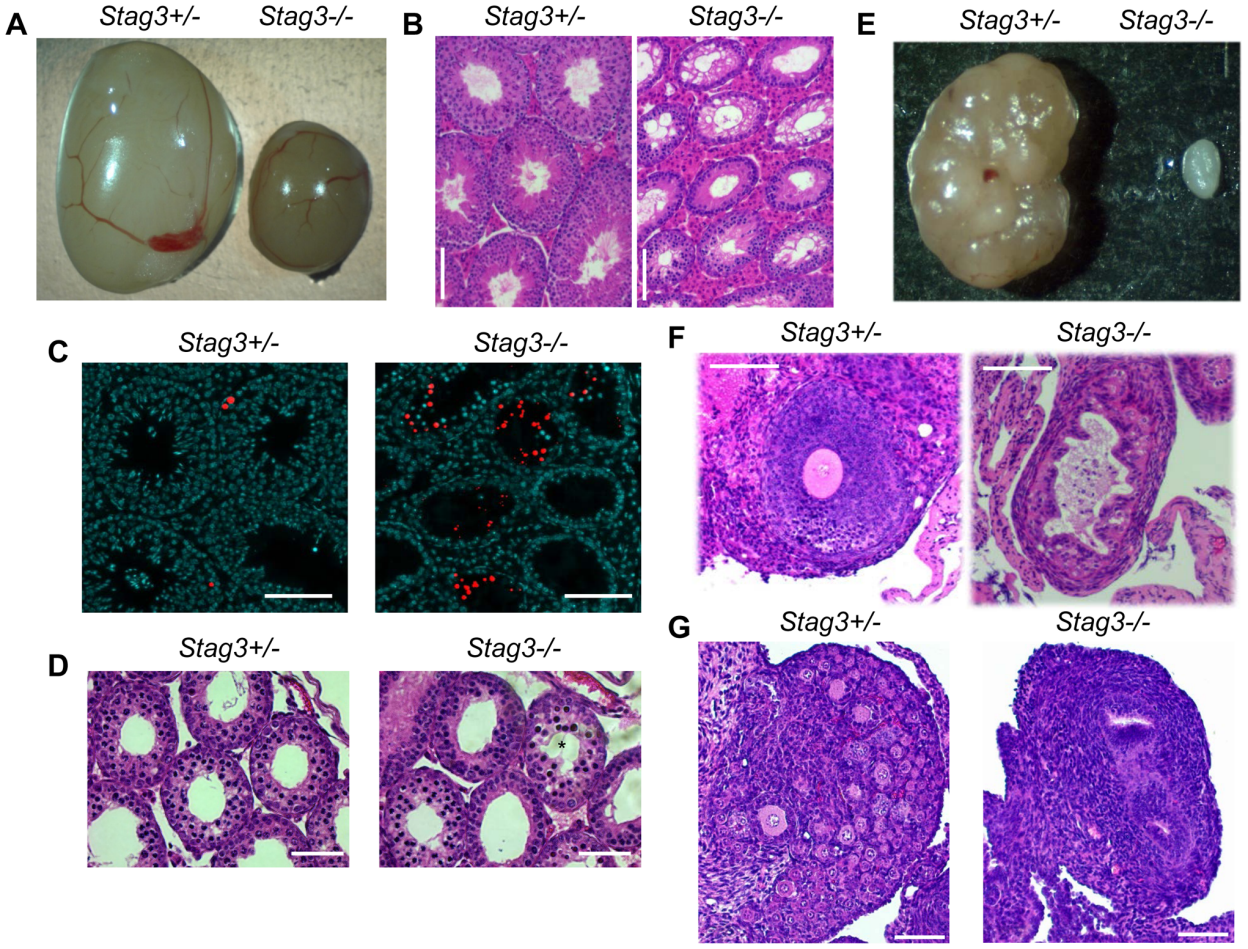
*Stag3* mutation results in gonadal failure. (A) Image of *Stag3^+/−^* and *Stag3^−/−^* testes at 8 weeks of age. The average testis to body weight ratio of six *Stag3^+/−^* and *Stag3^−/−^* 8 week old mice was 0.72% (+/− 0.05%) and 0.18% (+/− 0.02%) respectively. (B) Haemoxylin and eosin staining of 5 micron thick testis sections of 8 week old *Stag3^+/−^* and *Stag3^−/−^* mice, scale bar  = 100 µm. (C) TUNEL staining of paraffin embedded 5 micron thick testis sections of 8 week old *Stag3^+/−^* and *Stag3^−/−^* mice; scale bar  = 100 µm. (D) Haemoxylin and eosin staining of 5 micron thick testis sections of 18 days postpartum (dpp) *Stag3^+/−^* and *Stag3^−/−^* mice. The star represents a tubule that contains germ cells undergoing apoptosis, scale bar  = 100 µm. (E) Image of *Stag3^+/−^* and *Stag3^−/−^* ovaries at 8 weeks of age. The average ovary to body weight ratio of six *Stag3^+/−^* and *Stag3^−/−^* 8 week old mice was 0.044% (+/−0.0064%) and 0.0048% (+/−0.001%) respectively. (F) Haemoxylin and eosin staining of 5 micron thick ovary sections of 8 week old *Stag3^+/−^* and *Stag3^−/−^* mice; scale bar  = 50 µm. (G) Haemoxylin and eosin staining of 5 micron thick ovary sections of 6 dpp *Stag3^+/−^* and *Stag3^−/−^* mice, scale bar  = 100 µm. All images in this figure are from mice with the *Stag3^OV^* mutant allele.

We used two independently created *Stag3* mutant mouse lines, one created by lentiposon induced mutagenesis (*Stag3^Ov^* allele) and the other by targeted mutation (*Stag3^JAX^* allele, see Materials and Methods and Fig. S1). Mice homozygous for either mutation and mice containing a combination of both mutant alleles resulted in matching phenotypes with respect to fertility and meiotic defects (Table S1 and Fig. S2). Mice that were heterozygous for the *Stag3* mutations were phenotypically indistinguishable from their wild type littermates. Both female and male *Stag3* homozygous mutant mice were sterile (Table S1). For 8 week old *Stag3^Ov^* mutant mice, the average testis weight was 24.8% of their control litter mates (Fig. 1A, N = 6, SD = 1.77%). Testis sections stained with haemoxylin and eosin (H&E) showed a complete absence of secondary spermatocytes, round spermatids or elongated spermatids (Fig. 1B). Assessment of adult and juvenile testis sections with TUNEL and H&E staining showed that tubule degeneration was first evident during the first wave of spermatogenesis when mid-prophase I is reached (Fig. 1C and D). Spermatid counts from 30 day old mutant and control mice showed that no spermatids were present in the *Stag3* mutant tubules (106/1200 cells for heterozygote Vs 0/1200 for the *Stag3* mutant). In addition, sperm isolation from the epididymis of 80 day old mice showed that sperm were completely absent in the *Stag3* mutant. In 8 week old *Stag3^Ov^* mutant mice the average ovary weight was 10.9% of the size of their control litter mates (Fig. 1E, N = 6). H&E stained sections from adult and neonatal *Stag3* mutant ovaries showed the complete absence of oocytes (Fig. 1 F and G).

### *Stag3 mutation results in a zygotene-like stage arrest in male and female germ cells*

**Figure 2:**
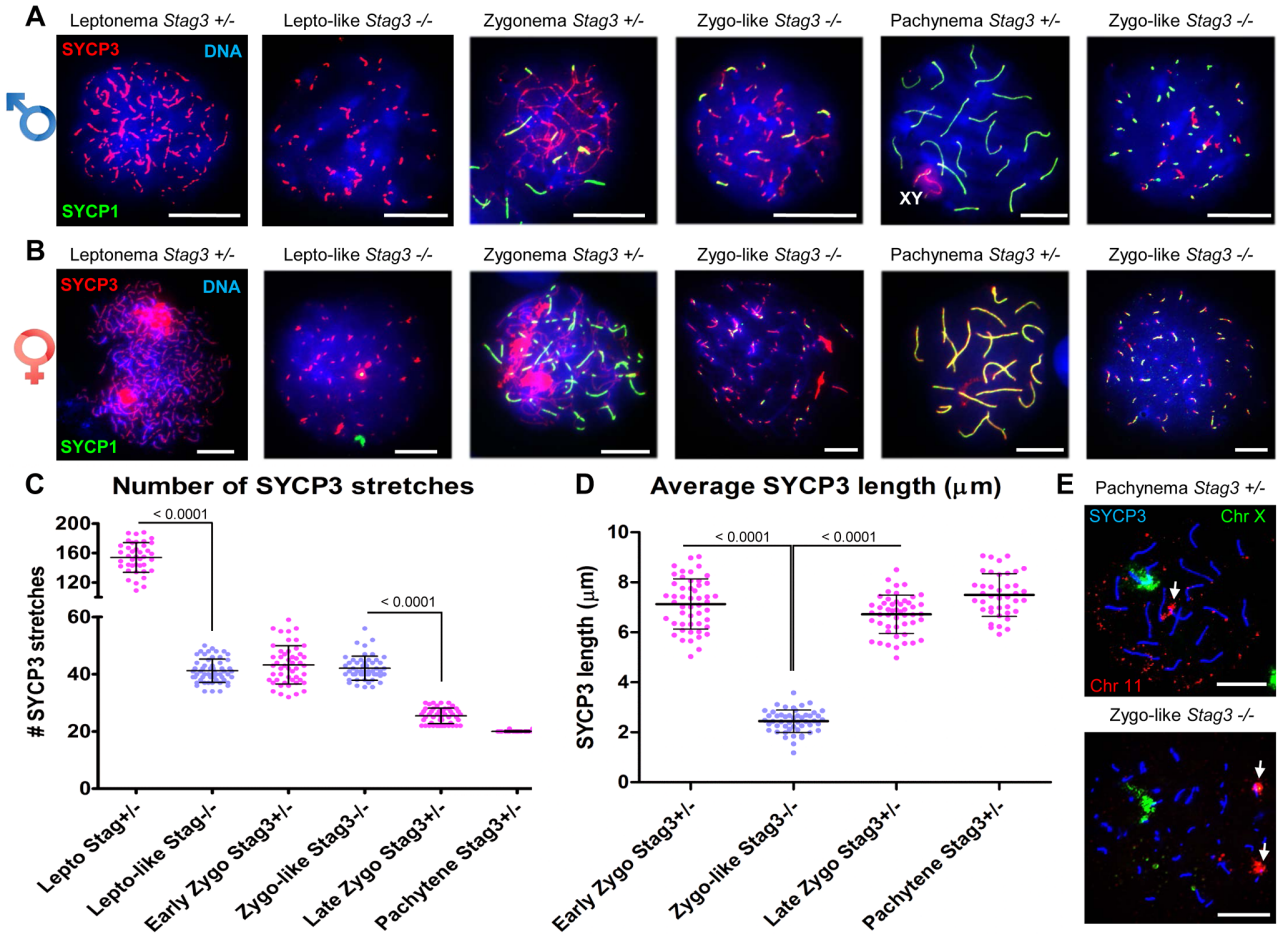
*Stag3* mutation results in abnormal meiosis progression, atypical synapsis between sister chromatids, and absence of pachytene stage germ cells. Chromatin spreads from (A) purified testicular germ cells of *Stag3^+/−^* and *Stag3^−/−^* mice aged 16 dpp and (B) embryonic ovarian germ cells of *Stag3^+/−^* and *Stag3^−/−^* mice aged 16.5 days post coitum were stained with DAPI (blue, DNA) and immunolabeled using antibodies against the SC lateral element protein SYCP3 (red) and the transverse filament of the central region of the SC SYCP1 (green). Meiotic prophase stages are indicated across the top; *Stag3^−/−^* spermatocytes and oocytes were deemed to be at a leptotene-like (lepto-like) stage when SYCP1 was not evident and at a zygotene-like stage (zygo-like) when SYCP1 colocalized with the SYCP3 signal. XY label represents the sex chromosome pair. Images in (A) and (B) are of spermatocytes carrying the *Stag3^OV^* mutant allele, but similar phenotypes were observed for spermatocytes with the *Stag3^JAX^* mutant allele and mice carrying the *Stag3^OV^* and *Stag3^JAX^* alleles combined (Fig. S2). (C) Scatter dot-plot graph of the number of SYCP3 linear stretches per spermatocyte chromatin spread during leptotene (lepto; average  = 154, N = 40), early zygotene (early zygo; average  = 43, N = 50), late zygotene (late zygo; average  = 25, N = 50) and pachytene (average  = 20, N = 40) stages for the *Stag3^+/−^* control and lepto-like (average  = 41, N = 50) and zygo-like (average  = 42, N = 51) stages for the *Stag3^−/−^* mice. Similar results were obtained when assessing oocyte chromatin spreads, summarized in Fig. S3. (D) Scatter dot-plot graph of the average SYCP3 length per spermatocyte chromatin spread during early zygo (7.1 µm), late zygo (6.7 µm) and pachytene (7.4 µm) stages for the *Stag3^+/−^* control and zygo-like (2.4 µm) stage for the *Stag3^−/−^* mice. Similar results were obtained when assessing oocyte chromatin spreads, summarized in Fig. S3. (E) Chromatin spreads from purified testicular germ cells of *Stag3^+/−^* and *Stag3^−/−^* mice aged 16 dpp were immunolabeled using an antibody against the SC lateral element protein SYCP3 (blue) and then hybridized to two pre-labelled FISH probes, one that detects the entire X chromosome (green) and the other detects 200 kilobases of mouse chromosome 11 (TK [11qE1]) distal to the centromere (red, white arrows). Mean and standard deviation of the columns of each graph are represented by the black bars and *P* values are given for indicated comparisons (Mann-Whitney, one-tailed). Experiments were performed using 4 separate littermate pairs of mutant and control mice. Scale bars  = 10 µm

Mouse mutants for all other meiosis-specific cohesin components display defects during meiotic prophase I in spermatocytes [16], [34], [36], [37]. To assess the meiotic defect of the *Stag3* mutants more closely, we assessed the formation of chromosome axes using immunofluorescence microscopy of spread chromatin. We staged the progression of prophase I using antibodies against axial/lateral element, SYCP3, and the central region protein SYCP1. *Stag3* male and female mutant primary germ cells show aberrancies in leptotene and zygotene stages and fail to reach the pachytene stage (Fig. 2 and Fig. S2). The leptotene stage in control spermatocytes is characterized by many short stretches of SYCP3 (axial elements between sister chromatids) and the absence of SYCP1 (Figure 2A and C; average for *Stag3^+/Ov^* control  = 154 SYCP3 stretches, N  = 40 nuclei). However, the *Stag3* mutants display a leptotene-like stage that has fewer SYCP3 stretches (Fig. 2A and C; average for *Stag3^Ov/Ov^* mutant  = 41 SYCP3 stretches, N = 69 nuclei). At the early zygotene stage, control spermatocytes display fewer, longer stretches of SYCP3, some of which colocalize with SYCP1 indicating that homologous chromosomes are beginning to synapse (Fig. 2A, C and D; average for *Stag3^+/Ov^* control  = 43 SYCP3 stretches, N = 50 nuclei). During later stages of zygotene, more extensive chromosome synapsis is evident and the number of SYCP3 stretches continues to decrease (Fig. 2A and C; average for *Stag3^+/Ov^* control  = 25.5 SYCP3 stretches, N = 50 nuclei). Finally, at the pachytene stage, autosomes of the control spermatocytes are completely synapsed and the XY chromosomes are paired within the sex body (Fig. 2A and C; average for *Stag3^+/Ov^* control  = 20 SYCP3 stretches, N = 40 nuclei). Chromatin spreads of the *Stag3* mutant spermatocytes showed SYCP1 loading and we consider these as a zygotene-like stage (Fig. 2A). However, as the extent of SYCP1 loading increased, the number of SYCP3 stretches did not decrease (Fig. 2A and C, most right panel; average for *Stag3^Ov/Ov^* mutant  = 42 SYCP3 stretches, N = 51 nuclei). Furthermore, the length of the SYCP3 stretches at the zygotene-like stage was approximately 66% shorter than the average length of SYCP3 stretches in wild type chromatin spreads (Fig. 2D). Similar differences in SYCP3 stretch length and number were measured between oocytes from control and *Stag3* mutant mice (Fig. 2B and Fig. S3).

Following pre-meiotic DNA replication, the number of sister chromatid pairs in mice is 40, which is similar to the number of SYCP3 stretches counted in prophase germ cells of the *Stag3* mutant (Fig. 2C). Therefore the SYCP1 loading observed in the zygotene-like chromatin spreads may represent sister chromatid synapsis. To determine whether this was the case we employed fluorescence in situ hybridization (FISH) using two fluorescently labelled DNA probes, one specific to 200 kb of chromosome 11 and the other to detect the X chromosome (Fig. 2E). In spermatocyte chromatin spreads from control mice staged at pachytene, only one FISH signal for each probe was observed. In contrast chromatin spreads from the *Stag3* mutant displayed two signals for chromosome 11. This suggests that the SYCP1 signals are indeed present on sister chromatids.

**Figure 3:**
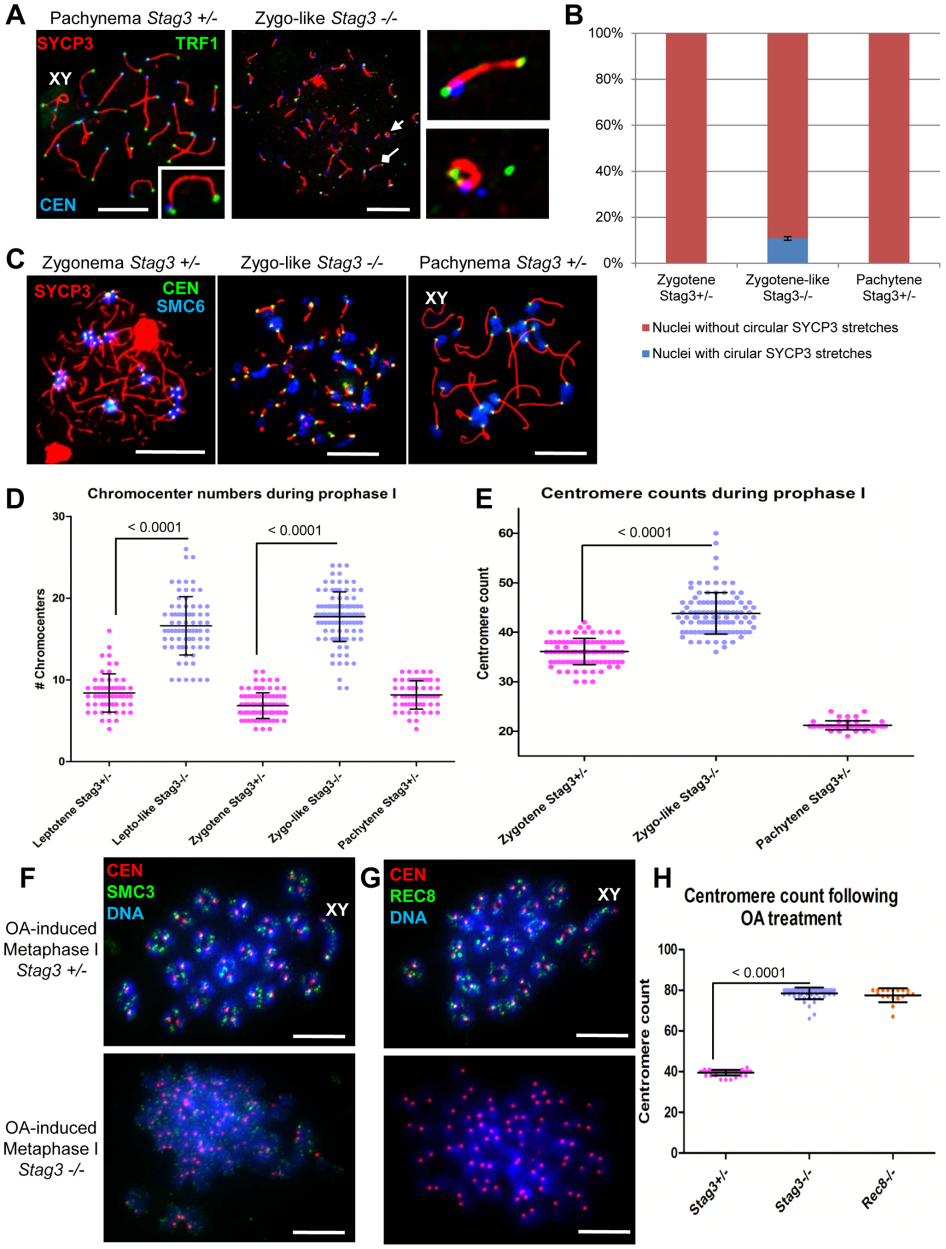
*Stag3* mutation results in circular SYCP3 stretches, disrupted heterochromatin pericentromeric clustering (chromocenters), and premature loss of centromere cohesion between sister chromatids. (A-E) Chromatin spreads were prepared from purified testicular germ cells of *Stag3^+/−^* and *Stag3^−/−^* mice aged 16 dpp. (A) Chromatin spreads were immunolabeled with antibodies against the SC lateral element protein SYCP3 (red), the centromere-kinetochore (blue, CEN) and the telomeric protein TRF1 (green). The left most panel is a *Stag3^+/−^* chromatin spread at pachytene stage. XY label represents the sex chromosome pair. Inset image on the bottom right corner is a 2× zoom of a synapsed autosome pair with a telomere signal at each end and a centromere signal at one end. The middle panel is a *Stag3^−/−^* chromatin spread at a zygo-like stage. The diamond and triangle arrow heads point to the SYCP3 stretches that are magnified in the right most panels. The top right most panel is a 5× zoom of a *Stag3^−/−^* SYCP3 stretch with a telomere signal at each end and a centromere signal at one end. The bottom right most panel is a 5× zoom of a *Stag3^−/−^* circular SYCP3 stretch. (B) Quantification of nuclei with circular SYCP3 stretches. No circular SYCP3 stretches were observed during zygotene or pachytene stages for the *Stag3^+/−^* control (N = 179 and 224 respectively), whereas 10.9% of zygo-like chromatin spreads from the *Stag3^−/−^* mice were recorded to have circular SYCP3 stretches (N = 212). This experiment was performed in triplicate and positive and negative error bars represent the highest and lowest percentage of circular SYCP3 stretches obtained. (C) Chromatin spreads were immunolabeled with antibodies against the SC lateral element protein SYCP3 (red), the centromere-kinetochore (green, CEN) and SMC6 protein which localizes to the pericentromeric heterochromatin clusters also known as “chromocenters” (blue). Meiotic prophase stages are indicated across the top. (D) Scatter dot-plot graph of the number of chromocenters per spermatocyte chromatin spread during leptotene (average  = 8.4, N = 56), zygotene (average  = 6.9, N = 89) and pachytene (average  = 8.2, N = 55) stages for the *Stag3^+/−^* control and lepto-like (average  = 16.6, N = 74) and zygo-like (17.7, N = 102) stages for the *Stag3^−/−^* mice. Similar results were obtained when assessing oocyte chromatin spreads, summarized in Fig. S4A and B. (E) Scatter dot-plot graph of the number of centromere-kinetochore signals per spermatocyte chromatin spread during zygotene (average  = 36.1, N = 89) and pachytene (average  = 21.2, N = 55) stages for the *Stag3^−/−^* mice and zygo-like stage (average  = 43.8, N = 102) for the *Stag3^−/−^* mice. Experiments were performed using 3 separate littermate pairs of mutant and control mice. Images are from germ cells carrying the *Stag3^OV^* allele, comparable phenotypes were observed for germ cells carrying the *Stag3^JAX^* mutant allele (Fig. S2). Similar results for centromere counts were obtained when assessing oocyte chromatin spreads summarized in Fig. S4A and C. (F and G) Chromatin spreads from purified and short-term cultured testicular germ cells of *Stag3^+/−^* and *Stag3^−/−^* mice aged 20 dpp following treatment with 5 µM of okadaic acid. (F) Chromatin spreads stained with DAPI (blue, DNA) and immunolabeled with antibodies against the SC lateral element protein SYCP3 (red) and the pan-cohesin component SMC3 (green). (G) Chromatin spreads stained with DAPI (blue, DNA) and immunolabeled with antibodies against the SC lateral element protein SYCP3 (red) and the meiosis-specific α-kleisin cohesin component REC8 (green). (H) Scatter dot-plot graph of the number of centromere-kinetochore signals per spermatocyte chromatin spread following 5 hours of OA treatment for *Stag3^+/−^* (average  = 39.5, N = 40), *Stag3^−/−^* (average  = 78.5, N = 60) and *Rec8^−/−^* (average  = 77.6, N = 18) mice. Mean and standard deviation of the columns of each graph are represented by the black bars and *P* values are given for indicated comparisons (Mann-Whitney, one-tailed). Scale bar  = 10 µm

Mouse chromosomes are telocentric, and STAG3, REC8 and RAD21L cohesins localize to the telomeres at the pre-leptotene stage of meiosis [15], [38]. To characterize the *Stag3* mutant chromosome axes further we assessed chromatin spreads immuno-stained for SYCP3, the centromere and telomeres (Fig. 3). In control chromatin spreads, a fully synapsed chromosome axis has a centromere and telomere signal at one end, and a telomere signal at the other (Fig. 3A). By analyzing chromatin spreads of the *Stag3* mutant, we determined that SYCP3 stretches can indeed form along the entire length of the chromosomes (Fig. 3A middle and top right panel). We also observed circular SYCP3 stretches that were not observed in the control (Fig. 3A bottom right panel and 3B). Circular SYCP3 structures have also been observed in *Smc1β* mutants and they may be the result of telomere fusion [17].

### Pericentromeric heterochromatin clustering is aberrant in a *Stag3* mutant

STAG3, REC8 and RAD21L cohesins also localize to the heterochromatin rich pericentromeric clusters (“chromocenters”) at the pre-leptotene stage of meiosis [3], [15]. In nuclear spread preparations chromocenters can be easily distinguished from the rest of the chromatin by their more dense DAPI staining and can be further confirmed by the presence of the centromeres and SMC5/6 components (Fig. 3C) [18], [19]. From analysis of leptotene stage chromatin spreads, it is evident that there are chromocenter associations between non-homologous chromosomes as there are on average 8.4 chromocenter bodies per nucleus (Fig. 3C and D, N = 56 nuclei). At this stage dynamic chromosome movements are occurring and it has been proposed that these chromocenter associations are important for initial chromosome pairing, DNA repair, and synapsis between homologues [13], [14]. At zygotene stage, chromocenter associations are even more apparent with an average of 6.9 chromocenter bodies per nucleus (Fig. 3C and D; N = 89 nuclei). In contrast the *Stag3* mutant shows reduced levels of chromosome associations within chromocenters at both leptotene-like and zygotene-like stages, showing on average 16.2 and 17.7 chromocenter bodies per nucleus respectively (Fig. 3C and D; N = 75 and 102 nuclei respectively). A similar trend for chromocenter counts was obtained from oocyte chromatin spreads of the *Stag3* mutant and controls (Fig. S4A and B). This result suggests that STAG3 plays a role in mediating early prophase associations of pericentromeric chromosome ends into chromocenters.

### Mutation of *Stag3* results in impaired centromere cohesion between sister chromatids

To count the number of centromere-kinetochore structures we used an anti-centromere autoantibody (CEN; also known as ACA and CREST). The average number of centromere-kinetochores counted in control zygotene and pachytene stage nuclei was 36.1 and 21.2 respectively (Figure 3C and E; N = 89 and 20 respectively), which corresponds well to the fact that the centromere-proximal ends are the last regions to synapse [39], [40]. In contrast the average number of centromeres counted in *Stag3* mutant zygotene-like nuclei was 43.8 (Fig. 3C and E, N = 102). The centromere number corresponds well with the number of SYCP3 signals observed in the *Stag3* mutant, also suggesting that synapsis is occurring between sister chromatids. In addition, 71% of zygotene-like *Stag3* mutant nuclei had greater than 40 centromeres, suggesting that centromere cohesion between sister chromatids is compromised (Fig. 3C and E). To further assess this possibility we exposed spermatocytes to okadaic acid (OA), which stimulates an artificial chromatin transition from prophase to metaphase I [41]. When wild type spermatocytes are exposed to OA, they form 20 bivalents each consisting of a centromere-kinetochore pair (40 centromeres, Fig. 3F-H, N = 40). Conversely, 80 separated centromere-kinetochore signals were observed for the *Stag3* mutant (N = 60), further demonstrating that STAG3 is required for centromere cohesion.

### Absence of STAG3 destabilizes meiosis-specific cohesins

From physical interaction studies, it has been shown that there are up to 6 cohesin complexes present during meiosis, 5 of which are meiosis-specific [3], [7], [8], [34]. SMC3 is the only subunit that is present within all cohesin complexes. From our OA treatment studies we determined that SMC3 remains present on the *Stag3* mutant chromatin (Fig. 3F), whereas REC8, a meiosis-specific kleisin subunit, was absent (Fig. 3G). This suggests centromere cohesion in this assay would also be lost in the absence of REC8, which was indeed the case (Fig. 3H).

**Figure 4:**
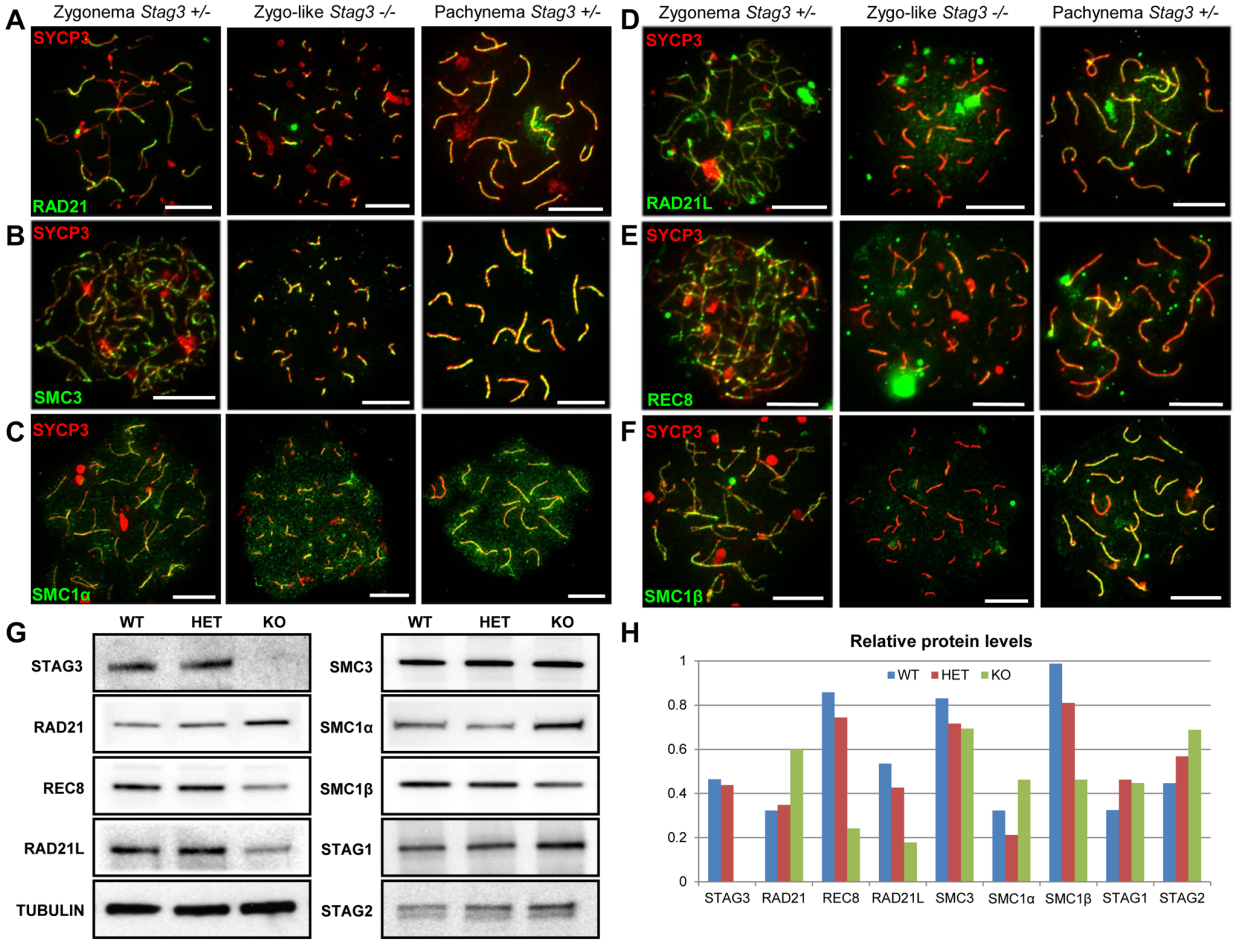
Mutation of *Stag3* does not affect the localization of components of the mitotic cohesin complex, but is required for the localization and stability of meiosis-specific cohesin subunits. Chromatin spreads were prepared from purified testicular germ cells of *Stag3^+/−^* and *Stag3^−/−^* mice aged 16 dpp. Chromatin spreads were immunolabeled with antibodies against the SC lateral element protein SYCP3 (red) and pan-cohesin component SMC3 (A), mitotic cohesin components SMC1α (B) and RAD21 (C) and meiosis-specific cohesin components RAD21L (D), REC8 (E) and SMC1β (F) in green. Meiotic prophase stages are indicated across the top. Experiments were performed using 3 separate littermate pairs of mutant and control mice. Images are from germ cells carrying the *Stag3^Ov^* allele. Similar results were obtained when assessing oocyte chromatin spreads, summarized in Fig. S5 and for the *Stag3^JAX^* allele mutants (Fig. S6). (G) Protein extracts from purified testicular germ cells of WT (*Stag3^+/+^*), HET (*Stag3^+/−^*) and KO (*Stag3^−/−^*) mice aged 16 dpp were prepared and western blot analyses performed for STAG3, RAD21, REC8, RAD21L, SMC3, SMC1α, SMC1β, STAG1 and STAG2. Tubulin was used as a loading control. (H) Quantification of protein levels of each cohesin component analyzed in (G). Tubulin was used to normalize the loading of each lane. Each western blot was repeated at least twice. Tubulin loading controls corresponding to each western blot analyzed is present in Fig. S7. Data shown for germ cell extracts from the *Stag3^OV^* homozygous mutants and littermate controls. Scale bar  = 10 µm

STAG3 is the only meiosis-specific cohesin subunit that is present in all of the meiosis-specific cohesins [3], [7], [8]. Using antibodies raised against both mitotic and meiosis-specific cohesins, we assessed whether the localization and protein levels of cohesin components were affected in the absence of STAG3. We observed the mitotic cohesin components RAD21, SMC3 and SMC1α colocalize with SYCP3 during the zygotene and pachytene stages of meiosis in wild type spermatocyte and oocyte chromatin spreads (Fig. 4A-C, S5A and B, S6A). These mitotic cohesin components also localize with SYCP3 in the chromatin spreads of the *Stag3* mutants. In addition, we immunoprecipitated SMC3 from germ cell extracts and assessed the co-immunoprecipitation of SMC1 and RAD21 (Fig. S7). From this we determined that the mitotic cohesin complex was not affected in the *Stag3* mutant. The meiosis-specific cohesin subunits, SMC1β, REC8 and RAD21L also colocalize with SYCP3 during the zygotene and pachytene stages of meiosis (Fig. 4D-F, S5C and D, S6B and C). Strikingly the colocalization of SMC1β, REC8 and RAD21L with SYCP3 in both male and female meiotic chromatin spreads are greatly reduced in the absence of STAG3.

Taking advantage of the nearly synchronous first wave of spermatogenesis, we purified germ cells from mice that are enriched for early stages of prophase I (16 days postpartum). Using protein extracts from these cells we assessed protein levels of cohesin subunits. We did not detect STAG3 protein in the *Stag3* mutant protein extracts. The *Stag3* mutant mice exhibited protein levels for mitotic cohesin subunits SMC3, SMC1α, STAG1 and STAG2 that were equivalent to control littermates (Fig. 4G and H and S8). However, levels of the mitotic kleisin subunit RAD21 were higher in the *Stag3* mutant. In contrast, levels of the meiosis-specific kleisin subunits RAD21L and REC8 were reduced in the *Stag3* mutant extracts. Furthermore, the meiosis-specific SMC1 protein, SMC1β was also reduced in the *Stag3* mutant extracts. From these observations it could be interpreted that STAG3 is required for the stability of the meiosis-specific cohesin components and is compensated for by an increase of mitotic cohesins in *Stag3* mutants. Another possible contributing factor is that levels of meiosis-specific cohesin components are lower due to meiotic arrest, and therefore an increased mitotic to meiotic germ cell ratio. Nevertheless, taken together with the nuclear spread data, we propose that STAG3 is required for the stability of meiosis-specific cohesins that are loaded onto chromosome axes.

### Mutation of *Stag3* results in the inability to repair SPO11-induced DSBs

**Figure 5:**
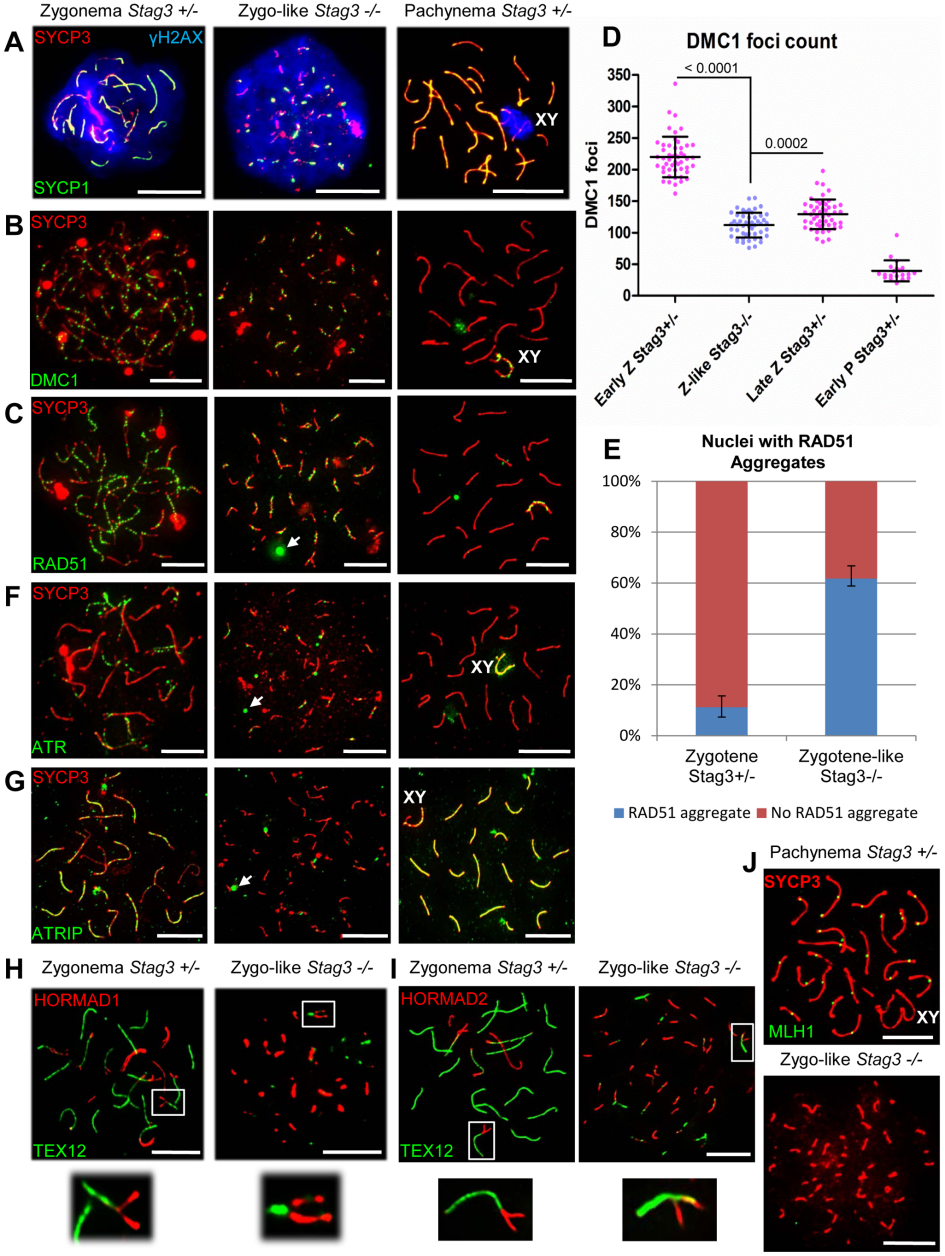
Stag3 mutants fail to repair meiotic DSBs and have an abnormal DNA damage response. Chromatin spreads from purified testicular germ cells of *Stag3^+/−^* and *Stag3^−/−^* mice aged 16 dpp were prepared and immunolabeled. (A) Chromatin spreads were immunolabeled with antibodies against the SC lateral element protein SYCP3 (red), phosphorylated histone H2AFX (blue, γH2AX) and the transverse filament of the central region of the SC SYCP1 (green). (B) Chromatin spreads were immunolabeled with antibodies against the SC lateral element protein SYCP3 (red) and meiosis-specific single-end invasion protein DMC1 (green). (C) Chromatin spreads were immunolabeled with antibodies against the SC lateral element protein SYCP3 (red) and single-end invasion protein RAD51 (green). Arrows represent RAD51 aggregates not associated with SYCP3 stretches. (D) Scatter dot-plot graph of the number of DMC1 foci per spermatocyte chromatin spread during early zygotene (Early Z, average  = 220, N = 50), late zygotene (Late Z, average  = 129, N = 50) and early pachytene (Early P, average  = 39.5, N = 20) stages for the *Stag3^+/−^* control and zygo-like stage (Z-like average  = 112, N = 50) for the *Stag3^−/−^* mice. Mean and standard deviation of each column of the graph are represented by the black bars and *P* values are given for indicated comparisons (Mann-Whitney, one-tailed). (E) Bar graph of the percentage of chromatin spreads that contain RAD51 aggregates at the zygotene stage (average  = 11.2%, N = 179) for the *Stag3^+/−^* control and zygotene-like stage (average  = 61.8%, N = 212) for the *Stag3^−/−^* mice. The error bars represent the variation between three independent experiments. (F) Chromatin spreads were immunolabeled with antibodies against the SC lateral element protein SYCP3 (red) and DNA damage response protein ATR (green). Arrows represent ATR aggregates not associated with SYCP3 stretches. (G) Chromatin spreads were immunolabeled with antibodies against the SC lateral element protein SYCP3 (red) and DNA damage response protein ATRIP (green). Arrows represent ATRIP aggregates. (H and I) Chromatin spreads were immunolabeled using antibodies against the HORMA domain containing protein HORMAD1 (H, red) or HORMAD2 (I, red) and the SC central element protein TEX12 (green). The boxed regions are magnified 3× below the whole chromatin spread images. Images are from the *Stag3^Ov^* mutant allele, comparable phenotype was observed for the *Stag3^JAX^* mutant allele (Fig. S2). (J) Chromatin spreads were immunolabeled with antibodies against the SC lateral element protein SYCP3 (red) and crossover protein MLH1 (green). Each experiment was performed at least twice. Images are from cells with the *Stag3^Ov^* mutant allele. XY label represents the sex chromosome pair. Scale bars  = 10 µm

To assess the DNA repair pathway, we examined whether the ATM/ATR mediated phosphorylation of H2AFX histones (γH2AX) was present in the *Stag3* mutants. In wild type spermatocyte chromatin spreads, γH2AX is widespread during the leptotene and zygotene stages (Fig. 5A). Following DNA repair the γH2AX signal is removed from the chromatin, remaining only on the X-Y chromatin by the pachytene stage (Fig. 5A). Chromatin spreads from male and female *Stag3* mutants showed that γH2AX was widespread throughout the chromatin, which suggests SPO11-induced DSBs are being formed and that a DNA damage response was activated (Fig. 5A and S9). However, the γH2AX signal is not removed from the chromatin, which suggests that the DNA damage is not repaired in *Stag3* mutants.

### *Stag3* mutants have a defective DNA damage response

To determine whether there are any defects in DNA repair by homologous recombination in meiosis, we assessed localization of the single-end invasion proteins, RAD51 and DMC1, in primary spermatocytes [42]. RAD51 and DMC1 load at DSB sites and promote DNA repair during zygonema (Fig. 5B and C). The number of DMC1 foci is highest during early zygotene stage for the control spermatocytes averaging 220 foci per nucleus (Fig. 5D, N = 50). DSBs begin to be repaired at the late zygotene stage and the average number of DMC1 foci reduces to 129 foci per nucleus (Fig. 5D, N = 50). DMC1 and RAD51 foci are mainly absent from the autosomes by early pachytene stage, but remain on the X-Y axes (Fig. 5B-D). DMC1 and RAD51 foci localized to the SYCP3 stretches in the *Stag3* mutant, however the numbers of DMC1 foci were lower in comparison to the early zygotene stage of the control (Fig 5B-D, 112 foci per nucleus, N = 50). Furthermore, DMC1 and RAD51 foci remained present on the SYCP3 stretches in the *Stag3* mutant, indicating that DSBs are not repaired. In addition, RAD51 aggregates were evident in more than 60% of the *Stag3* mutant chromatin spreads suggesting that DNA repair processes are aberrant (Fig. 5E). Together with the persistence of γH2AX, these observations show that SPO11-induced DSBs are not repaired in primary germ cells of the *Stag3* mutant.

It is known that ATR is responsible for a DNA damage checkpoint cascade which includes its interaction partner ATRIP [42]. During the zygotene stage, ATR-ATRIP signals the existence of recombination intermediates and activates the DNA damage response [24]. ATR localizes to unsynapsed regions of chromosome axes during zygonema, and then dissociate from the autosomes following synapsis (Fig. 5F) [43]. Unlike ATR and other ATR-mediated checkpoint proteins, ATRIP remains localized to the autosomes following synapsis (Fig5G) [24]. Localization of ATR and ATRIP to SYCP3 stretches in the *Stag3* mutant was aberrant, and often formed large aggregates that were not associated with SYCP3 (Fig. 5F and G).

HORMAD1 and 2 are asynapsis surveillance proteins preferentially localize to unsynapsed chromosome axes (Fig. 5H and I) [27]. Both proteins are required to stimulate regular levels of SPO11 induced DSBs and to trigger the ATR-mediated asynapsis response [23], [44]–[46]. Our data suggests that sister chromatids are synapsed in the *Stag3* mutant (Fig. 2). Therefore we wished to determine whether HORMAD1 and 2 proteins dissociate during this abnormal form of synapsis. We observed that the HORMAD proteins do dissociate from the synapsed regions of the chromosome axes (Fig. 5H and I), suggesting that the asynapsis surveillance mechanism does not distinguish between synapsis between homologues or sister chromatids.

In summary, meiotic DSBs formed in the *Stag3* mutant, and the DNA damage response mechanisms such as H2AFX phosphorylation, RAD51 and DMC1 loading were apparent. However, meiotic DSBs were not repaired in *Stag3* mutants and the ATR-mediated DNA damage response was abnormal.

## Discussion

### STAG3 - a conserved and essential meiosis-specific component

Stromal antigen (STAG) domain-containing cohesin subunits are common in eukaryotic model organisms including *Saccharomyces cerevisiae*, *Schizosaccharomyces pombe*, *Caenorhabditis elegans*, *Drosophila melanogaster* and mammals. Interestingly, there are meiosis-specific STAG domain proteins in a subset of these organisms. The fission yeast meiosis-specific STAG domain protein, Rec11 was shown to be a component of chromosome arm-specific cohesin with Rec8, whereas the mitotic STAG protein (Psc3) is a centromere cohesin component with Rec8 [47]. Rec11 cohesin is removed from the chromosome arms during the first meiotic division, whereas Psc3 cohesin remains until meiosis II. The localization pattern of STAG3 in primary spermatocytes is very similar to fission yeast Rec11, as STAG3 has been shown to localize to the axial/lateral elements during prophase and remains bound between sister chromatid arms at metaphase I [5]. The STAG3 arm cohesin is removed progressively from the arms during the metaphase to anaphase I transition, but a proportion of STAG3 remains in close proximity with the centromere until the onset of anaphase I during spermatogenesis [5]. However, the localization of STAG3 is sexually dimorphic, as it localizes between sister kinetochores from anaphase I to metaphase II in human oocytes [9]. Another meiosis-specific STAG protein is the Stromalin in Meiosis (SNM) protein of *Drosophila*. Surprisingly, SNM does not colocalize with SMC1, suggesting that its role is independent of cohesin [48]. In addition, SNM is specific to the male where meiosis is not coupled with homologue exchange, SC formation and chiasma formation [1]. SNM is required for linking achaismate homologous chromosomes during meiosis via “pairing sites” and ensures accurate chromosome segregation [48].

Here we have shown that mammalian *Stag3* is required for normal SC formation between homologous chromosomes and sister chromatid cohesion. Mutation of fission yeast *Rec11* resulted in impaired linear element formation and increased sister chromatid separation [49]. Furthermore, mutation of *Rec11* causes reduced levels of recombination [50]. Our study has shown that *Stag3* mutants are unable to form crossovers due to an inability to repair SPO11-induced meiotic DSBs. In summary, STAG3 and Rec11 have a number of similarities with respect to function during meiosis, whereas SNM is a divergent protein with unique functions specific to the *Drosophila* male. Nevertheless, each meiosis-specific STAG domain protein is essential for meiotic progression, and each has a conserved role in mediating pairing of homologous chromosomes.

### Common and unique characteristics of the meiosis-specific cohesin mutants

**Figure 6:**
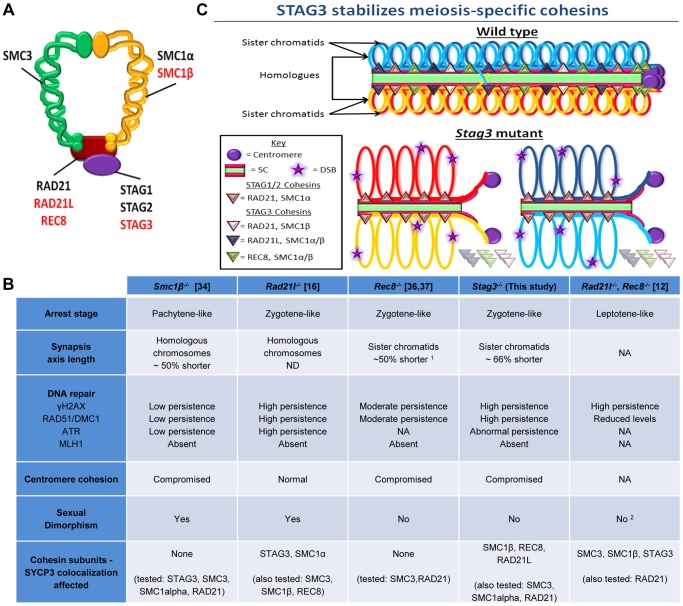
STAG3 is required for stability and loading of meiosis-specific cohesins to chromosome axes during meiosis. (A) Diagram depicting the ring-like structure of the cohesin complex. The mitotic cohesin and meiosis-specific components are written in black and red text respectively. (B) Summary table of the phenotypes recorded for mutants of the four meiosis-specific cohesin components. ^1^ The axis length was not defined in these studies, but from our analysis it is ∼50% shorter. ^2^ Although the female phenotype was not reported in this study, it can be implied due to the phenotype of the *Rec8^−/−^* mutant. (C) Cartoon of mid-prophase of a wild type (pachytene stage) and Stag3 mutant (zygotene-like stage). All features are described within the key. At pachytene stage, homologous chromosomes are fully synapsed and an obligate crossover has formed. In zygotene-like staged *Stag3* mutant germ cells, localization and stability of meiosis-specific cohesin complexes is aberrant and leads to synapsis between sister chromatids, DNA double strand breaks (DSBs) are not repaired and centromere cohesion between sister chromatids is perturbed. Chromatin loops are depicted to be larger in the *Stag3* mutant as their chromosome axes are shorter compared to wild type, and is supported by analysis of the *Smc1β* mutant mouse [70].

Four cohesin subunits are meiosis-specific in mammals, namely SMC1β, RAD21L, REC8 and STAG3 (Fig. 6A). There are up to six cohesin complexes associated with chromosomes during meiosis, including the mitotic cohesin (SMC1α-SMC3 bridged by STAG1 or 2 and RAD21), meiosis-specific SMC1β-containing cohesins (SMC1β-SMC3 bridged by STAG3 and either RAD21, REC8 or RAD21L) and meiosis-specific SMC1α-containing cohesins (SMC1α-SMC3 bridged by STAG3 and RAD21L or possibly REC8) [3], [7], [8], [34]. Therefore, STAG3 is the only component that is present in all meiosis-specific cohesins. By analyzing the *Stag3* mutant mouse, we have shown that STAG3 is required for stable localization of SMC1β, RAD21L and REC8 to chromosome axes, thus confirming their interaction in vivo.

Mutants of all four mouse meiosis-specific cohesin subunits have now been characterized using similar phenotype analyses such as meiotic progression, chromosome synapsis, DNA repair, centromere cohesion and localization of other cohesin components (Fig. 6B). In the male, mutation of each meiosis-specific cohesin component results in a prophase I arrest prior to crossover formation, nevertheless there are distinct features for each mutant. For instance, *Smc1β* mutation results in a pachytene-like stage arrest with a majority of chromosome synapsis and DNA repair occurring between homologues [34]. However, the synapsed chromosomes in a *Smc1β* mutant are shorter than in wild type and it was demonstrated that chromosome loops are larger. Mutation of the α-kleisin, *Rad21l*, gives rise to an arrest at a zygotene-like stage where homologous chromosomes are partially synapsed, but there is also a degree of non-homologous synapsis and SPO11-induced DSBs are not efficiently repaired [16]. In contrast, mutation of the other meiosis-specific α-kleisin, *Rec8*, results in synapsis between sister chromatids and although still aberrant, DNA repair is more apparent [36, 37, unpublished data]. *Rad21l*, *Rec8* double mutant spermatocytes arrest at a leptotene-like stage where the SYCP3 protein forms aggregates, showing that these α-kleisin subunits are both important for axial element formation [12]. The *Stag3* mutation results in a zygotene-like stage arrest similar to the *Rec8* mutant where sister chromatids are synapsed. However, the phenotype is more pronounced in the *Stag3* mutant, with chromosome axis length being shorter and the level of residual DNA damage being greater (Fig. 6B and C). In addition, the *Stag3* mutation caused the formation of circular SYCP3 axes, which are conceivably the result of telomere fusion, a phenomenon also observed in the *Smc1β* mutant [17]. Our data also suggests that STAG3 is required for maintenance of centromere cohesion between sister chromatids, which is a function shared by SMC1β and REC8, but not RAD21L [16], [34], [51]. If STAG3 is a component of all meiosis-specific cohesins, why is the *Stag3* mutant phenotype less pronounced than the *Rad21l*, *Rec8* double mutant? Based on the structure of cohesin complexes, the V-shaped heterodimer formed by SMC1 and SMC3 is bridged by one of the α-kleisins, and the STAG proteins interact with the α-kleisin (Fig. 6A) [52], [53]. Therefore, STAG proteins may not be required for cohesin ring formation per se, but required for cohesin ring stability (Fig. 6C). Our data supports this hypothesis as we observe a degree of SMC1β, REC8 and RAD21L loading onto the chromosome axes in the *Stag3* mutant, whereas there is complete absence of STAG3 loading in germ cells of the *Rad21l*, *Rec8* double mutant [12]. Also, the DNA damage response defect observed for the *Rad21l*, *Rec8* double mutant is more severe than the *Stag3* mutant (Fig 6B). Knockdown of single SMC complex components in tissue culture experiments has been shown to result in the decrease in protein stability of other components of the same cohesin complex. For example, RNAi mediated knockdown of SMC3 results in reduced SMC1 and RAD21 protein levels [54]. SMC1β, REC8 and RAD21L protein levels are decreased in a *Stag3* mutant, which further supports the hypothesis that STAG3 is required for the stability of meiosis-specific cohesins. We also observed higher levels of mitotic cohesin components in germ cell protein extracts of the *Stag3* mutant, particularly RAD21. It is possible that mitotic cohesins compensate for the loss of REC8 and RAD21L cohesin complexes in the *Stag3* mutant, which may not be the case in the *Rad21l*, *Rec8* double mutant. Mutational analyses combining the *Stag3* mutant with mutants of the other meiosis-specific cohesin subunits and conditional mutants for mitotic cohesin components will help test our hypothesis further.

Mutants of all four mouse meiosis-specific cohesin subunits also display differing phenotypes in the female germline (Fig. 6B). The *Rad21l* mutant phenotype is the least severe, with females exhibiting subfertility after 6 months of age and become prematurely infertile [16]. While oocytes of the *Smc1β* mutants progress to metaphase II, they are grossly aneuploid [34]. On the other hand, both female *Stag3* and *Rec8* mutants display a phenotype analogous to that observed in males [36], [37]. This suggests that STAG3-REC8 cohesin complexes are the predominant cohesin required for meiotic progression in females. Heterozygous mutations for *Smc1β* and *Rec8* have been reported to give rise to increased levels of premature sister chromatid separation in oocytes from adult mice [55]. It would certainly be interesting to determine whether this is the case for *Stag3* heterozygote mutants.

### Homologue recognition and cohesins

Homologue recognition/association initiates upon entry into meiotic prophase, prior to DSB formation and axis assembly [13], [14], [32]. As repetitive elements constitute 30–50% of the mammalian genome, it has been proposed that large chromosome elements such as sub-telomeric regions and peri-centromeric heterochromatin are crucial for initial homologue recognition [14]. Mouse chromosomes are telocentric, and it has been demonstrated that the peri-centromeric heterochromatin accumulates at the nuclear envelope during pre-leptotene and form clusters known as “chromocenters” [19]. During meiosis, STAG3, REC8 and RAD21L localize to the telomeres and chromocenters at pre-leptotene [3], [8], [15]. To facilitate initial pairing during pre-leptotene, telomere ends attach to the nuclear envelope. The notion that cohesins are required to stabilize these telomere attachments is supported by the fact that this event is partially defective in *Smc1β* and *Rad21l* mutants [16], [17]. Furthermore, it was demonstrated that STAG3 cohesins stabilize telomere attachment to the nuclear envelope via interaction with the telomere TRF1-TERB1 protein complex [15]. The TRF1-TERB1 protein complex also interacts with the nuclear membrane protein complex, SUN-KASH, which is required for stimulating chromosome movements that promotes chromosome pairing/synapsis [56]. In future work, it will be very interesting to assess the effect of the *Stag3* mutation on telomere binding to the nuclear envelope.

Although telomere movement is important for facilitating efficient chromosome pairing/synapsis, a recent report showed that it is not essential for the initial stages of homologue recognition [32]. The researchers of this study found that RAD21L is required for DSB-independent homologue recognition, and proposed that initial homologue pairing is based on homology of chromosome architecture. As STAG3 is a component of the RAD21L cohesins, it is very likely that STAG3 is required for the initial homologue recognition. We have shown that mutation of *Stag3* results in the dramatic decrease in pericentromeric heterochromatin clustering during meiotic prophase. The pericentromeric heterochromatin clustering phenomenon occurs at the same time as the initial homologue recognition [14], [32]. As STAG3 is required to ensure normal chromocenter formation, and STAG3-RAD21L localize to chromocenters, we propose that pericentromeric heterochromatin is a component of the chromosome architecture required for the initial homologue recognition. In addition, REC8 localizes to the pericentromeric chromatin. We have shown that STAG3-REC8 cohesins are required for maintaining sister chromatid cohesion. Robust sister chromatid cohesion at metaphase I may also require loading of cohesins to the chromocenters at meiotic entry. This is supported by the fact that cohesin loading at pericentromeric heterochromatin is important for maintenance of sister chromatid cohesion during mitosis [57], [58].

### Cohesinopathies – meiotic-specific components

Cohesinopathies is a term coined to encompass all human disorders caused by mutations in genes encoding for cohesin components or cofactors [59]. Mutations in RAD21, SMC1α and SMC3 have been shown to result in Cornelia de Lange syndrome, which causes intellectual disability and growth retardation and as well as facial and limb anomalies [60]–[62]. Based on mouse studies, it has also been suggested that *Stag1* mutation can cause Cornelia de Lange syndrome [63]. These disorders are attributed to the role of cohesin in regulating gene expression via interaction with CCCTC-binding factor sites or with mediator proteins, which repress or enhance gene expression respectively [59]. It is conceivable that expression of meiosis-specific cohesin subunits in mitotic cells could also give rise to cohesinopathies and cancer. For example, it has been shown that p53 mutated lymphoma cells express *Rec8* and *Stag3* [64] and an allele of *Stag3* is linked with the development of epithelial ovarian cancer [65]. Furthermore, it was recently shown that an inherited mutation in human *Stag3* that gives rise to infertility and gonadal failure [66].

### Conclusion

Using two independently derived mutations of mouse *Stag3*, we have determined that STAG3 is essential for fertility. Mutation of *Stag3* causes a zygotene-like meiotic prophase I arrest in both males and females. We show that STAG3 is required for the localization of the meiosis-specific subunits of cohesin, SMC1β, RAD21L and REC8, to chromosomal axes during meiotic prophase. STAG3 cohesins are required for DNA repair of SPO11-induced DSBs, synapsis between homologues, centromeric cohesion between sister chromatids, and heterochromatin-rich pericentromeric clustering between non-homologous chromosomes to form chromocenters.

## Materials and Methods

### Ethics statement

All mice were bred by the investigators at The Jackson Laboratory (JAX, Bar Harbor, ME) and Johns Hopkins University (JHU, Baltimore, MD) under standard conditions in accordance with the National Institutes of Health and U.S. Department of Agriculture criteria and protocols for their care and use were approved by the Institutional Animal Care and Use Committee (IACUC) of The Jackson Laboratory and Johns Hopkins University.

### Mice

Two mutations for *Stag3* were used in this study. 1) 1–8 cell stage FVB/N embryos were mutated by random insertion of the SB-cHS4core-SB-Tyro-WPRE-FUGW lentiposon transgene (LV2229). Using inverse PCR analysis, the lentiviral integration site was identified in intron 8 of the stromal antigen 3 gene (Stag3) on chromosome 5. The 3'-LTR is linked to the (+) strand of DNA at position 138,735,815 bp [NCB137/mm9; 3'-138,735,815(+)]. The lentivirus is inserted in the sense orientation relative to the disrupted mouse gene (Fig. S1A, http://www.mmrrc.org/catalog/sds.php?mmrrc_id=36275). The resulting heterozygote mice (FVB/N-Stag3TgTn(sb-cHS4,Tyr)2312COve/Mmjax) were bred together to create homozygote offspring which were compared to heterozygote and wild type littermate controls. 2) C57BL/6N-derived JM8.N4 embryonic stem (ES) cells that were targeted with a *β-galactosidase* containing cassette that generated a knockout first reporter allele for *Stag3* that harbored a floxed exon 5 were sourced from the International Knockout Mouse Consortium [67], http://www.knockoutmouse.org/martsearch/project/22907). As part of the KOMP2 program (http://commonfund.nih.gov/KOMP2/) these ES cells were injected into B6(Cg)- Tyrc-2J/J blastocysts. The resulting chimeric males were bred to C57BL/6NJ females and then to B6N.Cg-Tg(Sox2-cre)1Amc/J mice to remove the floxed neomycin and exon 5 (Fig. S1B). Offspring were bred to C57BL/6NJ mice or to wildtype siblings to remove the cre-expressing transgene resulting in the heterozygote B6N(Cg)-Stag3tm1b(KOMP)Wtsi/2J strain used in this study. Offspring homozygous for the Stag3tm1b(KOMP)Wtsi/2J allele were compared to heterozygote and wild type littermate controls. To confirm the phenotypes, heterozygote B6N(Cg)-Stag3tm1b(KOMP)Wtsi/2J animals were bred to FVB/N-Stag3TgTn(sb-cHS4,Tyr)2312COve/Mmjax to create experimental offspring that harbored both alleles, which were compared to heterozygote offspring for either allele. The *Rec8* mutant mice used in our study has previously been described [36].

### Histological analysis and TdT-mediated dUTP nick end labelling (TUNEL) assay

Testis and ovary tissues were fixed in bouins fixative. Tissues were embedded in paraffin and serial sections 5 microns thick were placed onto slides and stained with hematoxylin and eosin. For the TUNEL assay, sections were deparafinnized and apoptotic cells were detected using the in situ BrdU-Red DNA fragmentation (TUNEL) assay kit (Abcam) and counterstained with DAPI.

### Mouse germ-cell isolation and culture

Isolation of mixed germ cells from testes was performed using techniques previously described [68], [69]. Germ cells isolated from 16 day old male mice enriched for mid-prophase spermatocytes (2.5×10^6^ cells/ml) were cultured for 10 hr at 32°C in 5% CO2 in HEPES (25 mM)-buffered MEMα culture medium (Sigma) supplemented with 25 mM NaHCO3, 5% fetal bovine serum (Atlanta Biologicals), 10 mM sodium lactate, 59 µg/ml penicillin, and 100 µg/ml streptomycin. To initiate the G2/MI transition, cultured pachytene spermatocytes were treated with 5 µM okadaic acid (OA) (CalBiochem).

### Protein analyses

For protein level analyses, proteins were extracted from germ cells using RIPA buffer (Santa Cruz) containing 1× protease inhibitor cocktail (Roche). Protein concentration was calculated using a BCA protein assay kit (Pierce). Lanes of 4–15% gradient SDS polyacrylamide gels (Bio-Rad) were loaded with 20 µl of 1 mg/ml protein extract. Following protein separation via standard SDS PAGE, proteins were transferred to PVDF membranes using the Trans-Blot® Turbo™ western transfer system (Bio-Rad). Primary antibodies and dilution used are presented in Supplemental Table S2. At a 1∶20,000 dilution, Invitrogen horseradish peroxidase-conjugated antibodies rabbit anti-mouse (R21455), goat anti-rabbit (A10533), rabbit anti-goat (R21459) were used as secondary antibodies. The presence of antibodies on the PVDF membranes was detected via treatment with Pierce ECL western blotting substrate (Thermo Scientific) and captured using the Syngene XR5 gel documentation system. Protein levels were assessed using Image J (NIH). The SMC3 Co-IP experiment was performed using the Dynabead® Co-IP kit (Life Technologies). Each milligram of beads was covalently linked to 4 µg of SMC3 antibody (Abcam, ab9263) or corresponding IgG control antibody (Life Technologies, A10533).

### Spread chromatin analyses

Germ cell chromatin spreads were prepared as previously described [19], [31]. Primary antibodies and dilution used are presented in Supplemental Table S2. Secondary antibodies against human, rabbit, rat, mouse and guinea pig IgG and conjugated to Alexa 350, 488, 568 or 633 (Life Technologies) were used at 1∶500 dilution. Chromatin spreads were mounted in Vectashield + DAPI medium (Vector Laboratories). For fluorescence in situ hybridization (FISH), we used a pre-labelled FISH probes, one probe was used to detect 200 kilobases of mouse chromosome 11 (TK [11qE1]) distal to the centromere, and the other to recognize the X chromosome (Creative Bioarray). Prior to performing FISH, nuclear spreads were immuno-stained with rabbit anti-SYCP3 followed by the corresponding secondary conjugated to Alexa 633. We performed FISH following the manufacturer's protocol for cell preparations. Briefly, slides were incubated in 10 mM sodium citrate (pH 6.0) at 96°C in for 15 min, dehydrated and air dryed. The FISH probes and chromatin spreads were co-denatured at 80°C for 10 min under a 22×22 mm coverslip sealed with Fixogum (Marabu GmbH & Co.). Following hybridization at 37°C overnight slides were washed in 0.4×SSC+0.1% Igepal for 2 min then 2×SSC+0.3% Igepal for 1 min. Slides were dehydrated and mounted in Vectashield. Nuclear spread images were captured using a Zeiss CellObserver Z1 linked to an ORCA-Flash 4.0 CMOS camera (Hamamatsu) and analyzed with the Zeiss ZEN 2012 blue edition image software including foci and length measurement capabilities and Photoshop (Adobe) was used to prepare figure images.

## Supporting Information

Figure S1 Two *Stag3* mutants used for this study. (A) *Stag3^Ov^* mutant allele: 1-8 cell stage FVB/N embryos were mutated by random insertion of the SB-cHS4core-SB-Tyro-WPRE-FUGW lentiposon transgene (LV2229). See the Materials and Methods section for further information. (B) *Stag3^JAX^* mutant allele: C57BL/6N-derived JM8.N4 embryonic stem (ES) cells that were targeted with a *β-galactosidase* containing cassette that generated a knockout first reporter allele for *Stag3* that harbored a floxed exon 5 were sourced from the International Knockout Mouse Consortium. See the Materials and Methods section for further information.
(PDF)

Figure S2 Assessment of the *Stag3^JAX^* allele mutants confirms the phenotype described for the *Stag3^Ov^* allele mutants. (A) Spermatocyte chromatin spread preparations of *Stag3^JAX^* control and mutant were immunolabeled using antibodies against the SC lateral element protein SYCP3 (red) and the transverse filament of the central region of the SC SYCP1 (green). (B) Oocyte chromatin spread preparations of *Stag3^JAX^* control and mutant were immunolabeled using antibodies against the SC lateral element protein SYCP3 (red) and the transverse filament of the central region of the SC SYCP1 (green). (C) Spermatocyte chromatin spread preparations of *Stag3^JAX^* control and mutant were immunolabeled using antibodies against the SC lateral element protein SYCP3 (red), HORMA domain containing protein HORMAD1 (blue) and the SC central element protein TEX12 (green). (D) Oocyte chromatin spread preparations of *Stag3^JAX^* control and mutant were immunolabeled using antibodies against the SC lateral element protein SYCP3 (red), the transverse filament of the central region of the SC SYCP1 (green) and the centromere-kinetochore (blue, CEN). (E) Spermatocyte chromatin spread preparations of *Stag3^JAX^* heterozygote control and *Stag3^JAX/Ov^* mutant were immunolabeled using antibodies against the SC lateral element protein SYCP3 (red), HORMA domain containing protein HORMAD2 (blue) and the SC central element protein TEX12 (green). (F) Oocyte chromatin spread preparations of *Stag3^JAX^* heterozygote control and *Stag3^JAX/Ov^* mutant were immunolabeled using antibodies against the SC lateral element protein SYCP3 (red), the transverse filament of the central region of the SC SYCP1 (green) and the centromere-kinetochore (blue, CEN). Images are representative of the most advanced stage of meiosis observed in prophase germ cells of the *Stag3* mutants. Meiotic prophase stages are indicated above each panel column. Scale bars = 10 µm
(PDF)

Figure S3 Quantification of SYCP3 stretch number and length in mouse oocytes. (A) Scatter dot-plot graph of the number of SYCP3 linear stretches per oocyte chromatin spread during pachytene (average  = 20, N = 20) stage for the *Stag3^+/−^* control and zygo-like (average  = 42.5, N = 20) stage for the *Stag3^−/−^* mice. (B) Scatter dot-plot graph of the average SYCP3 length per spermatocyte chromatin spread during pachytene (7.7 µm) stage for the *Stag3^+/−^* control and zygo-like (2.5 µm) stage for the *Stag3^−/−^* mice. Mean and standard deviation of the columns of each graph are represented by the black bars and *P* values are given for indicated comparisons (Mann-Whitney, one-tailed).
(PDF)

Figure S4 Quantification of pericentromeric heterochromatin clusters (“chromocenters”) and centromeres in *Stag3* control and mutant mouse oocytes. (A) Chromatin spreads were immunolabeled with antibodies against the SC lateral element protein SYCP3 (red), the centromere-kinetochore (green, CEN) and SMC6 protein which localizes to the pericentromeric heterochromatin clusters also known as “chromocenters” (blue). Meiotic prophase stages are indicated across the top. (B) Scatter dot-plot graph of the number of chromocenters per oocyte chromatin spread during zygotene (average  = 14, N = 40) stage for the *Stag3^+/−^* control and zygo-like (20.3, N = 40) stage for the *Stag3^−/−^* mice. (C) Scatter dot-plot graph of the number of centromere-kinetochore signals per oocyte chromatin spread during zygotene (average  = 36.4, N = 40) and stage for the *Stag3^−/−^* mice and zygo-like stage (average  = 44.7, N = 40) for the *Stag3^−/−^* mice. Mean and standard deviation of the columns of each graph are represented by the black bars and *P* values are given for indicated comparisons (Mann-Whitney, one-tailed). Scale bars  = 10 µm
(PDF)

Figure S5 Mutation of *Stag3* results in aberrant localization of meiosis-specific cohesins in oocytes. Oocyte chromatin spreads immunolabeled with antibodies against the SC lateral element protein SYCP3 (red) and (A) SMC3, (B) RAD21, (C) REC8 and (D) RAD21L (green). Meiotic prophase stages are indicated across the top. Scale bars  = 10 µm
(PDF)

Figure S6 Assessment of the *Stag3^JAX^* allele mutants confirms the aberrant localization of meiosis-specific cohesins described for the *Stag3^Ov^* allele mutants. Spermatocyte chromatin spread preparations of *Stag3^JAX^* control and mutant were immunolabeled using antibodies against the SC lateral element protein SYCP3 (red) and (A) RAD21, (B) RAD21L and (C) REC8 (green). Meiotic prophase stages are indicated across the top. Scale bars  = 10 µm
(PDF)

Figure S7 *Stag3* mutation does not affect mitotic cohesin complex formation. Germ cell protein extracts from 8 week old *Stag3^+/−^* and *Stag3^−/−^* mice were used for immunoprecipitation with an antibody raised against SMC3 (A). The elute from both *Stag3^+/−^* and *Stag3^−/−^* extracts showed successful co-immunoprecipitation of cohesin component SMC1 (B).
(PDF)

Figure S8 *Stag3* mutation causes reduction in meiosis specific cohesin subunit protein levels. Western blots for STAG3 and STAG2 (A), STAG1 and SMC1β (B), REC8 (C), RAD21L and SMC1α (D), SMC3 and RAD21 (E) and their corresponding tubulin loading controls.
(PDF)

Figure S9 Mutation of *Stag3* causes a failure to repair DSBs during meiosis in oocytes. Oocyte chromatin spreads immunolabeled with antibodies against the SC lateral element protein SYCP3 (red) and γH2AX (blue). Meiotic prophase stages are indicated across the top. Scale bars  = 10 µm
(PDF)

Table S1 Fertility tests for *Stag3* mutants and controls. Each mouse was mated to wild type mice of corresponding backgrounds, until at least two rounds of pups were produced for the control mice. *Stag3* mutant and control males were mated to two wild type females. *Stag3* mutant and control females were mated to a single wild type male.
(PDF)

Table S2 Primary antibodies used in this in this study. Animal host, source, catalogue number (where applicable), and dilution for immunofluorescence microscopy or western blot are listed.
(PDF)
