# Supplementary figures and images for: Meiosis-Specific Cohesin Component, Stag3 Is Essential for Maintaining Centromere Chromatid Cohesion, and Required for DNA Repair and Synapsis between Homologous Chromosomes
Source: PLoS Genet. 2014 Jul 3;10(7):e1004413. doi: 10.1371/journal.pgen.1004413 (PMC4081007; doi:10.1371/journal.pgen.1004413)

# A

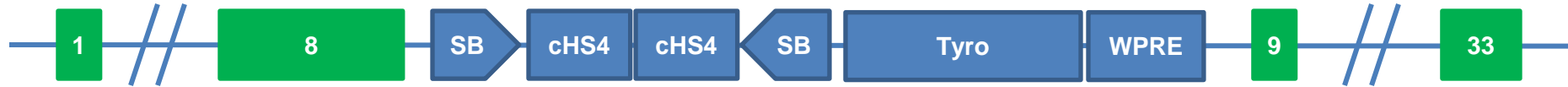

# B

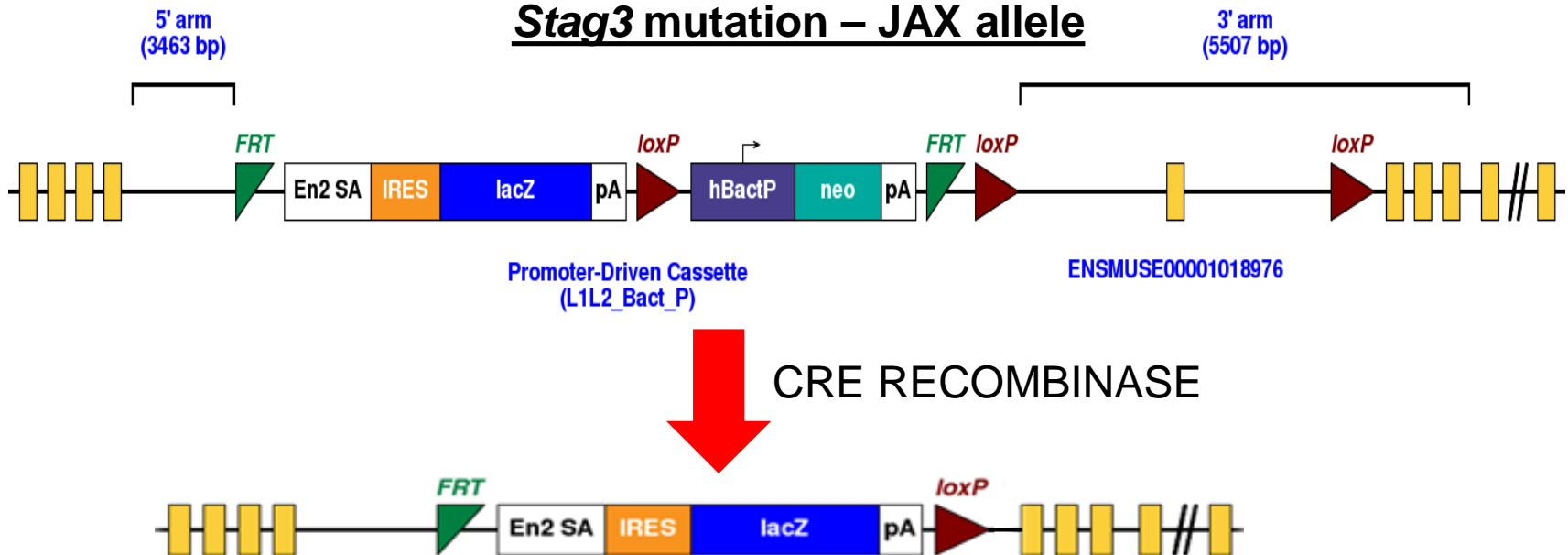

Supplement: Figure S1 — Two Stag3 mutants used for this study. (A) Stag3Ov mutant allele: 1-8 cell stage FVB/N embryos were mutated by random insertion of the SB-cHS4core-SB-Tyro-WPRE-FUGW lentiposon transgene (LV2229). See the Materials and Methods section for further information. (B) Stag3JAX mutant allele: C57BL/6N-derived JM8.N4 embryonic stem (ES) cells that were targeted with a β-galactosidase containing cassette that generated a knockout first reporter allele for Stag3 that harbored a floxed exon 5 were sourced from the International Knockout Mouse Consortium. See the Materials and Methods section for further information. (PDF) [file pgen.1004413.s001.pdf]

## **Stag3 mutation – JAX allele**

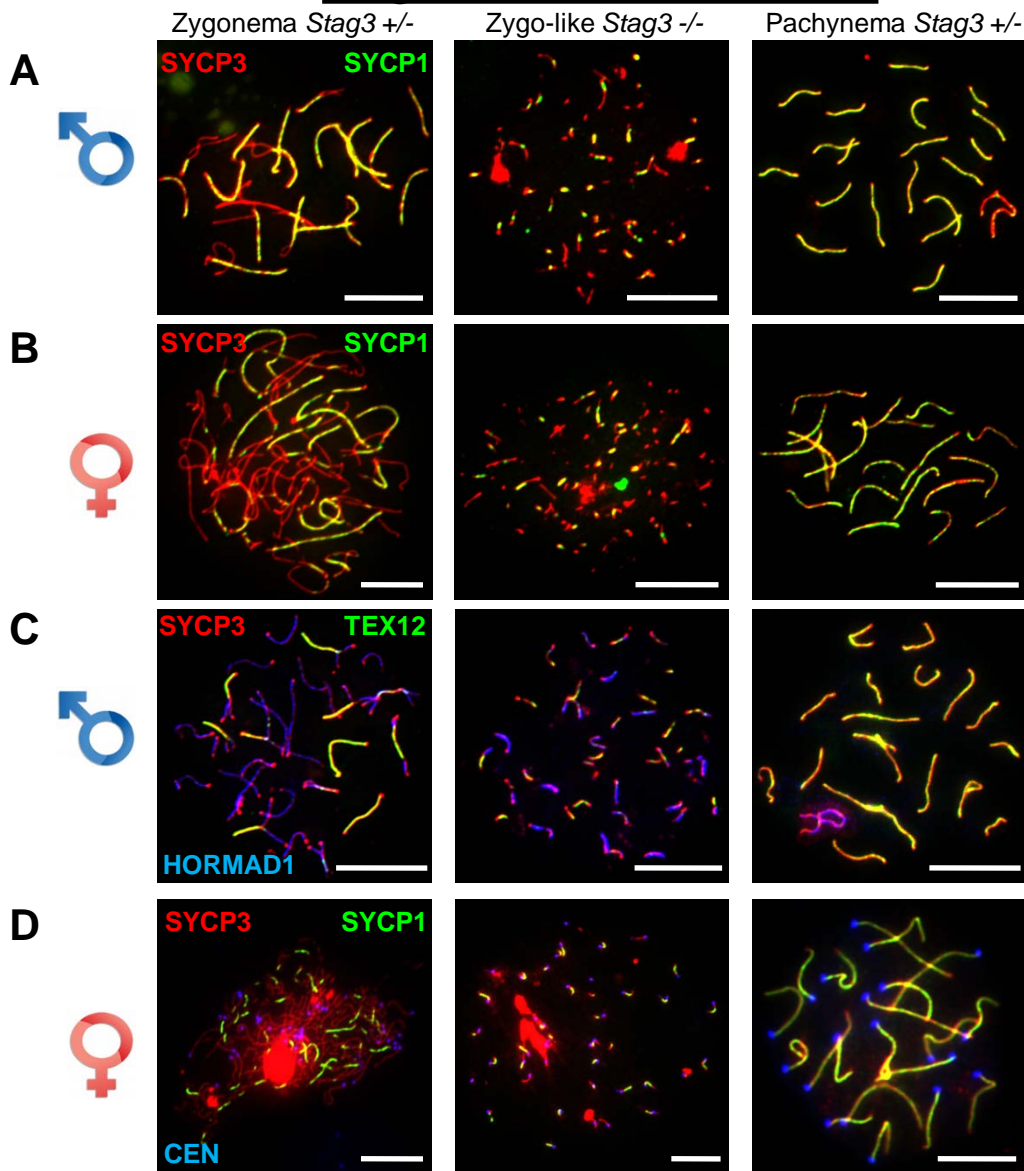

## **Stag3 mutation – JAX and Ov alleles combined**

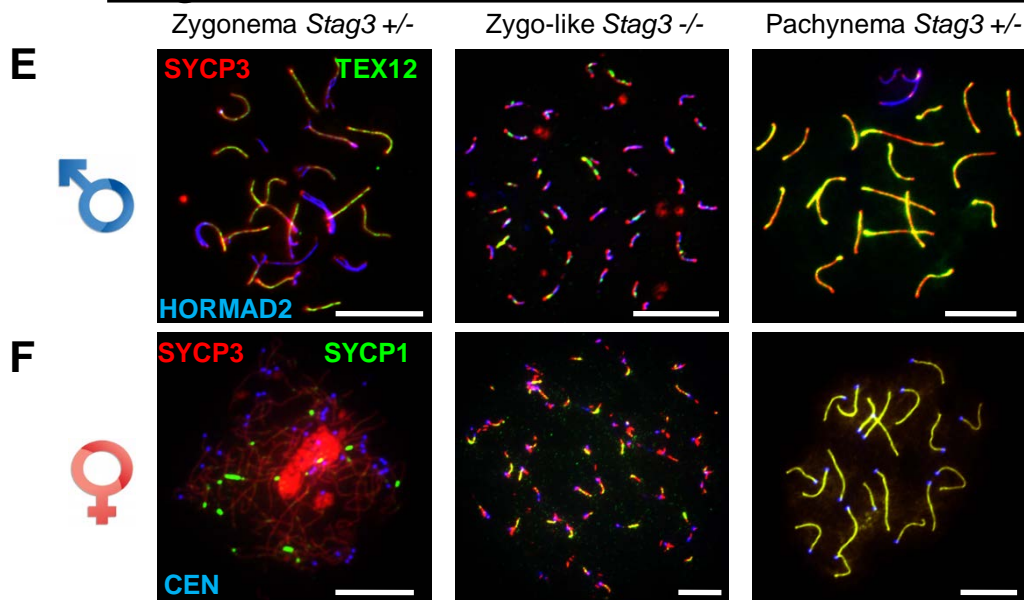

Supplement: Figure S2 — Assessment of the Stag3JAX allele mutants confirms the phenotype described for the Stag3Ov allele mutants. (A) Spermatocyte chromatin spread preparations of Stag3JAX control and mutant were immunolabeled using antibodies against the SC lateral element protein SYCP3 (red) and the transverse filament of the central region of the SC SYCP1 (green). (B) Oocyte chromatin spread preparations of Stag3JAX control and mutant were immunolabeled using antibodies against the SC lateral element protein SYCP3 (red) and the transverse filament of the central region of the SC SYCP1 (green). (C) Spermatocyte chromatin spread preparations of Stag3JAX control and mutant were immunolabeled using antibodies against the SC lateral element protein SYCP3 (red), HORMA domain containing protein HORMAD1 (blue) and the SC central element protein TEX12 (green). (D) Oocyte chromatin spread preparations of Stag3JAX control and mutant were immunolabeled using antibodies against the SC lateral element protein SYCP3 (red), the transverse filament of the central region of the SC SYCP1 (green) and the centromere-kinetochore (blue, CEN). (E) Spermatocyte chromatin spread preparations of Stag3JAX heterozygote control and Stag3JAX/Ov mutant were immunolabeled using antibodies against the SC lateral element protein SYCP3 (red), HORMA domain containing protein HORMAD2 (blue) and the SC central element protein TEX12 (green). (F) Oocyte chromatin spread preparations of Stag3JAX heterozygote control and Stag3JAX/Ov mutant were immunolabeled using antibodies against the SC lateral element protein SYCP3 (red), the transverse filament of the central region of the SC SYCP1 (green) and the centromere-kinetochore (blue, CEN). Images are representative of the most advanced stage of meiosis observed in prophase germ cells of the Stag3 mutants. Meiotic prophase stages are indicated above each panel column. Scale bars = 10 µm (PDF) [file pgen.1004413.s002.pdf]

**A**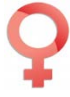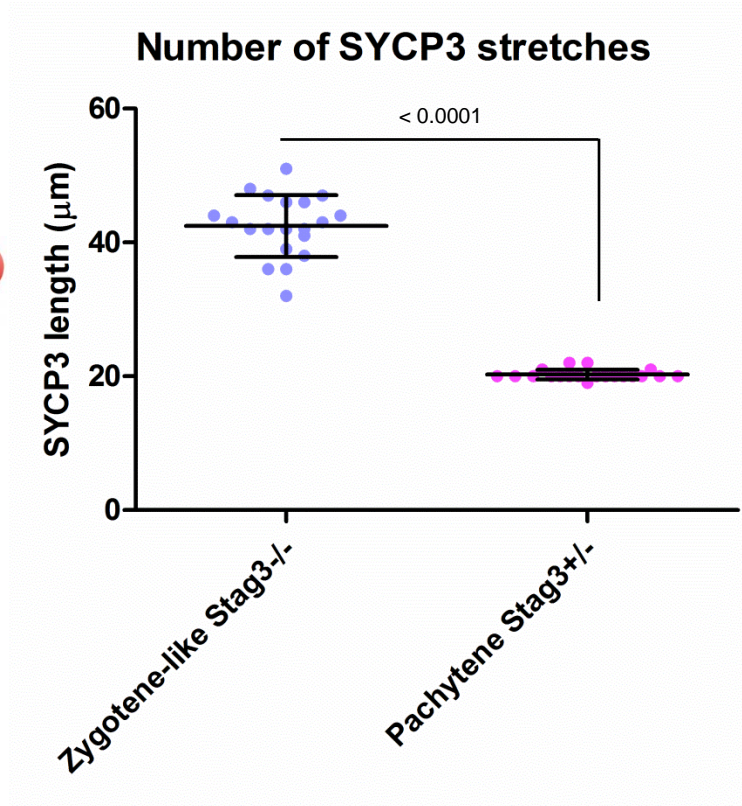**B**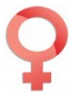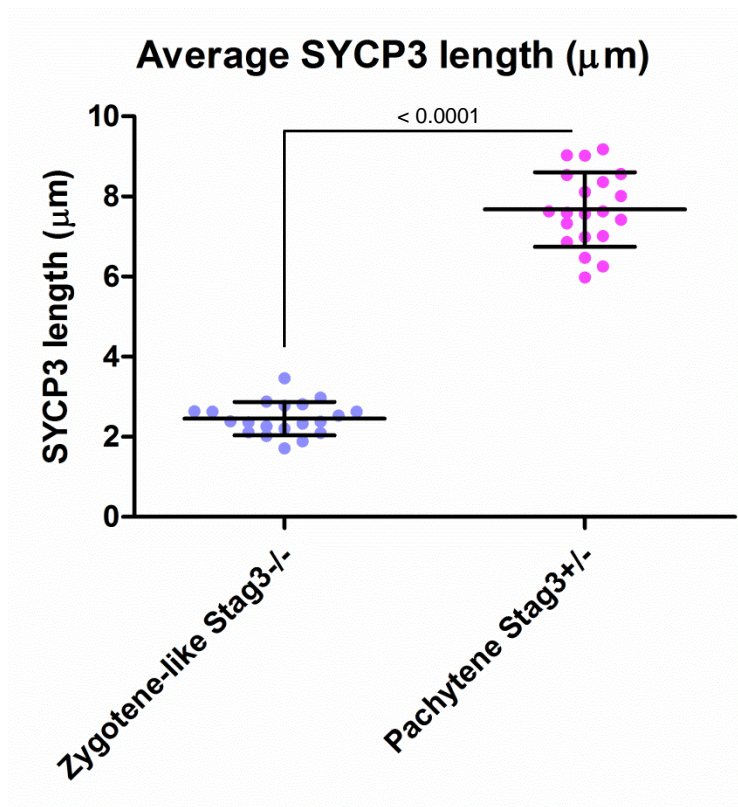

Supplement: Figure S3 — Quantification of SYCP3 stretch number and length in mouse oocytes. (A) Scatter dot-plot graph of the number of SYCP3 linear stretches per oocyte chromatin spread during pachytene (average = 20, N = 20) stage for the Stag3+/− control and zygo-like (average = 42.5, N = 20) stage for the Stag3−/− mice. (B) Scatter dot-plot graph of the average SYCP3 length per spermatocyte chromatin spread during pachytene (7.7 µm) stage for the Stag3+/− control and zygo-like (2.5 µm) stage for the Stag3−/− mice. Mean and standard deviation of the columns of each graph are represented by the black bars and P values are given for indicated comparisons (Mann-Whitney, one-tailed). (PDF) [file pgen.1004413.s003.pdf]

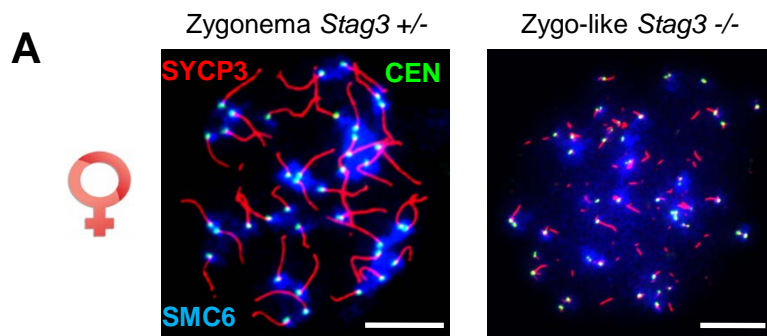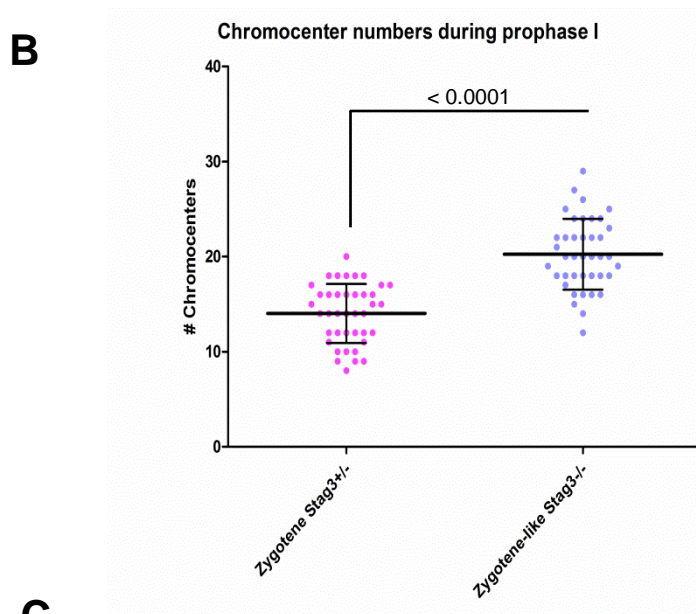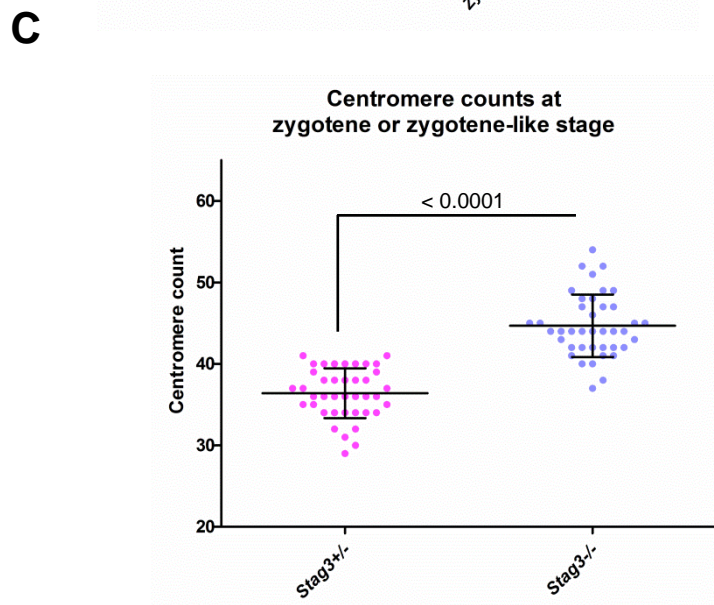

Supplement: Figure S4 — Quantification of pericentromeric heterochromatin clusters (“chromocenters”) and centromeres in Stag3 control and mutant mouse oocytes. (A) Chromatin spreads were immunolabeled with antibodies against the SC lateral element protein SYCP3 (red), the centromere-kinetochore (green, CEN) and SMC6 protein which localizes to the pericentromeric heterochromatin clusters also known as “chromocenters” (blue). Meiotic prophase stages are indicated across the top. (B) Scatter dot-plot graph of the number of chromocenters per oocyte chromatin spread during zygotene (average = 14, N = 40) stage for the Stag3+/− control and zygo-like (20.3, N = 40) stage for the Stag3−/− mice. (C) Scatter dot-plot graph of the number of centromere-kinetochore signals per oocyte chromatin spread during zygotene (average = 36.4, N = 40) and stage for the Stag3−/− mice and zygo-like stage (average = 44.7, N = 40) for the Stag3−/− mice. Mean and standard deviation of the columns of each graph are represented by the black bars and P values are given for indicated comparisons (Mann-Whitney, one-tailed). Scale bars = 10 µm (PDF) [file pgen.1004413.s004.pdf]

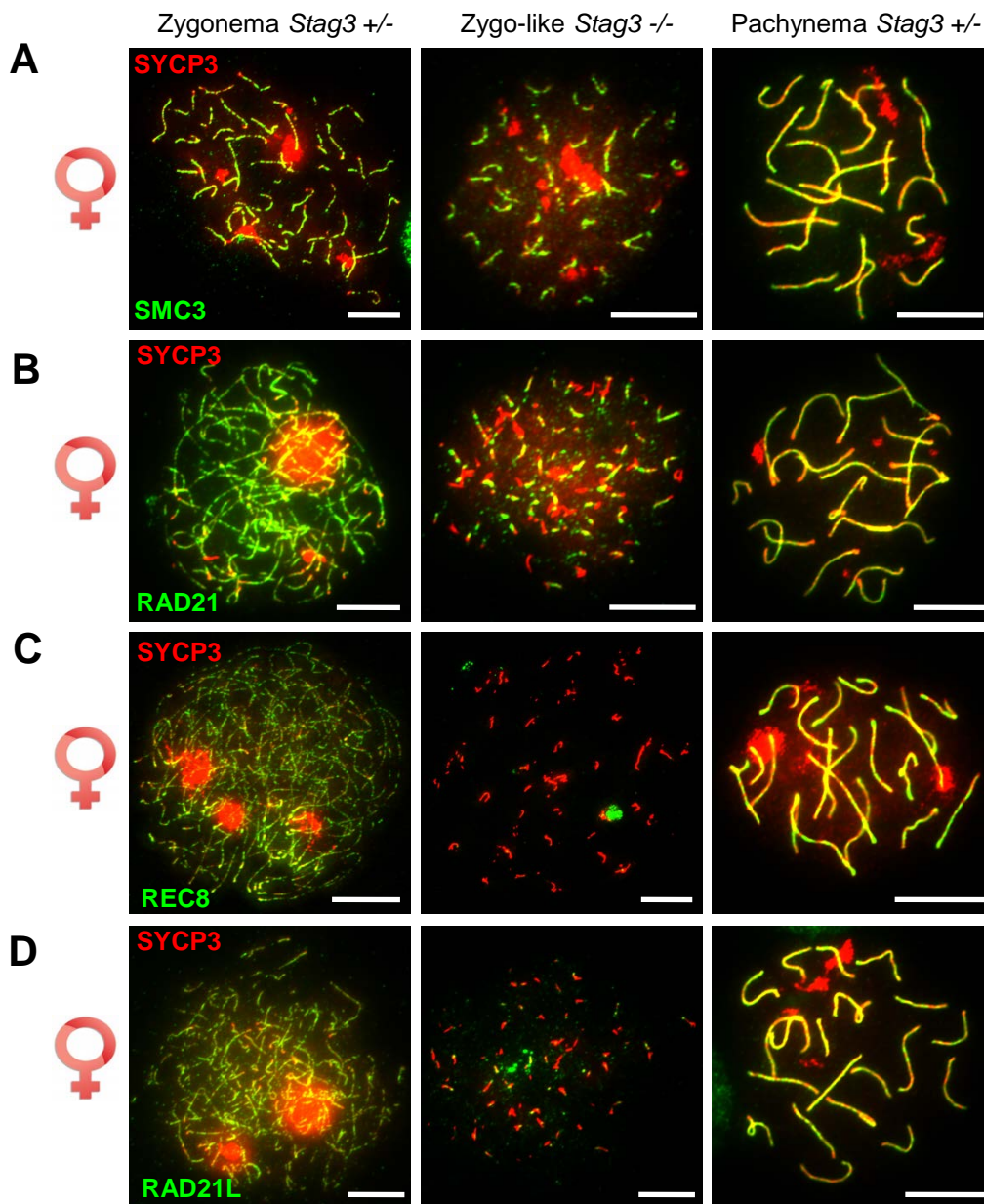

Supplement: Figure S5 — Mutation of Stag3 results in aberrant localization of meiosis-specific cohesins in oocytes. Oocyte chromatin spreads immunolabeled with antibodies against the SC lateral element protein SYCP3 (red) and (A) SMC3, (B) RAD21, (C) REC8 and (D) RAD21L (green). Meiotic prophase stages are indicated across the top. Scale bars = 10 µm (PDF) [file pgen.1004413.s005.pdf]

## **Stag3** mutation – JAX allele

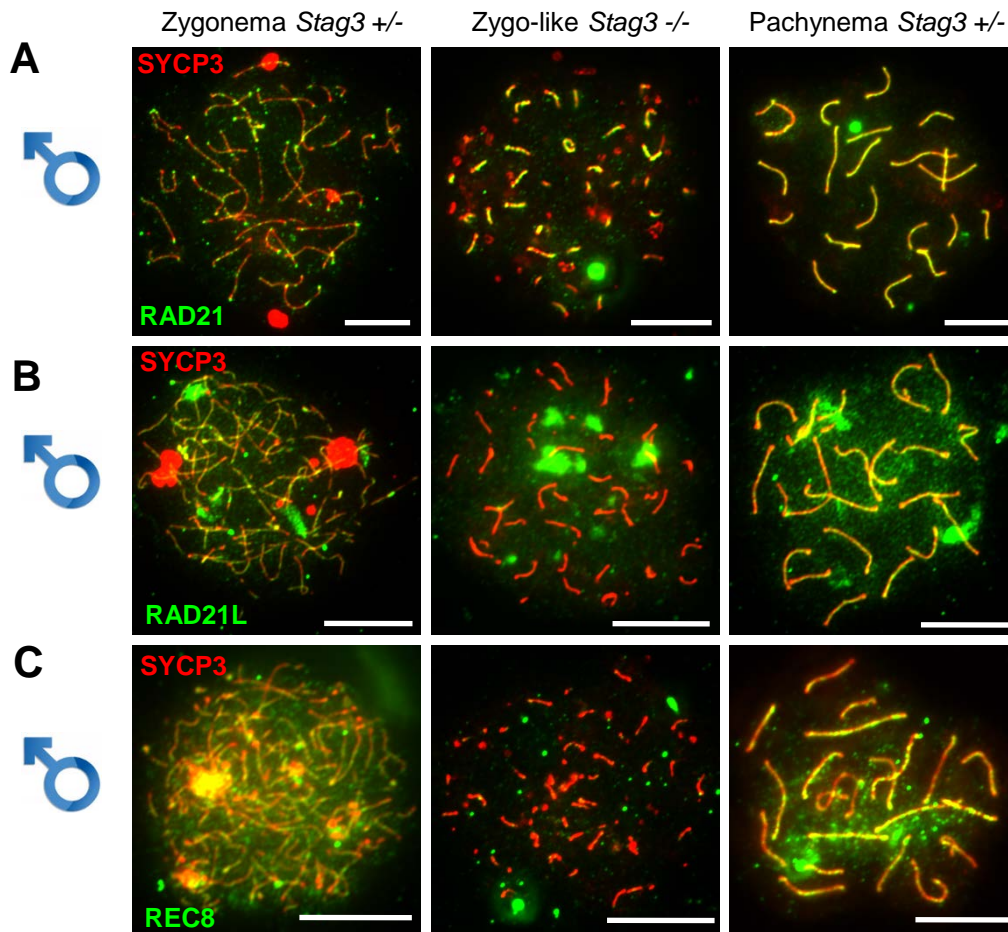

Supplement: Figure S6 — Assessment of the Stag3JAX allele mutants confirms the aberrant localization of meiosis-specific cohesins described for the Stag3Ov allele mutants. Spermatocyte chromatin spread preparations of Stag3JAX control and mutant were immunolabeled using antibodies against the SC lateral element protein SYCP3 (red) and (A) RAD21, (B) RAD21L and (C) REC8 (green). Meiotic prophase stages are indicated across the top. Scale bars = 10 µm (PDF) [file pgen.1004413.s006.pdf]

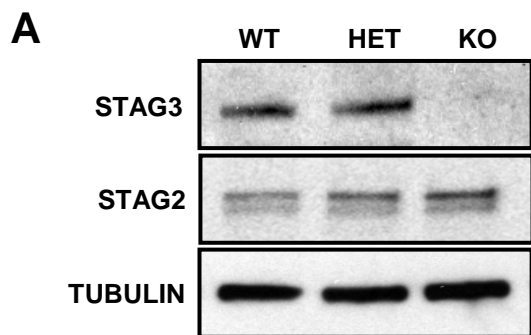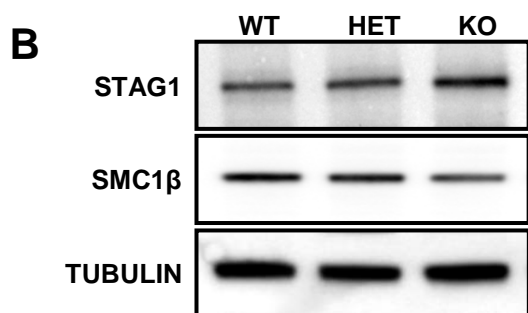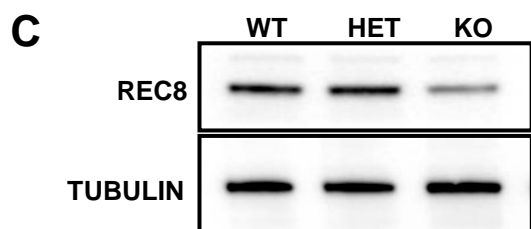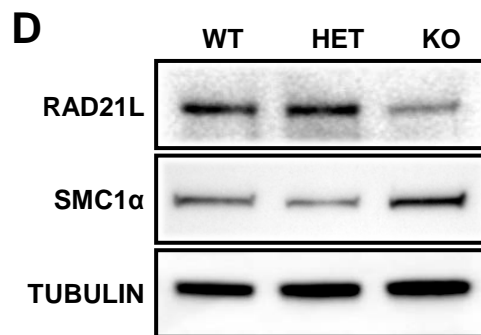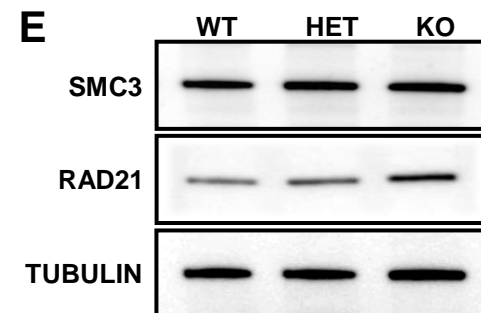

Supplement: Figure S8 — Stag3 mutation causes reduction in meiosis specific cohesin subunit protein levels. Western blots for STAG3 and STAG2 (A), STAG1 and SMC1β (B), REC8 (C), RAD21L and SMC1α (D), SMC3 and RAD21 (E) and their corresponding tubulin loading controls. (PDF) [file pgen.1004413.s008.pdf]

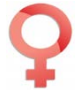

Zygonema *Stag3* +/-

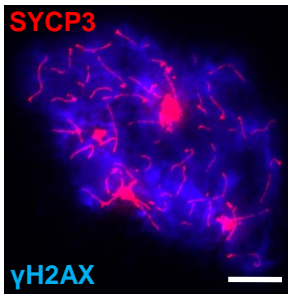

Zygo-like *Stag3* -/-

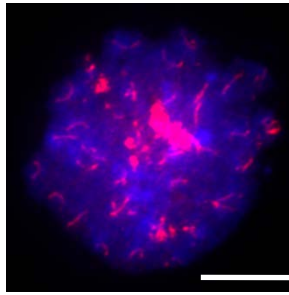

Pachynema *Stag3* +/-

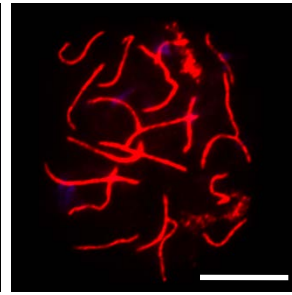

Supplement: Figure S9 — Mutation of Stag3 causes a failure to repair DSBs during meiosis in oocytes. Oocyte chromatin spreads immunolabeled with antibodies against the SC lateral element protein SYCP3 (red) and γH2AX (blue). Meiotic prophase stages are indicated across the top. Scale bars = 10 µm (PDF) [file pgen.1004413.s009.pdf]
