# Supplementary material for: Meiosis-Specific Cohesin Component, Stag3 Is Essential for Maintaining Centromere Chromatid Cohesion, and Required for DNA Repair and Synapsis between Homologous Chromosomes
Source: PLoS Genet. 2014 Jul 3;10(7):e1004413. doi: 10.1371/journal.pgen.1004413 (PMC4081007; doi:10.1371/journal.pgen.1004413)
Supplement: Figure S7 — Stag3 mutation does not affect mitotic cohesin complex formation. Germ cell protein extracts from 8 week old Stag3+/− and Stag3−/− mice were used for immunoprecipitation with an antibody raised against SMC3 (A). The elute from both Stag3+/− and Stag3−/− extracts showed successful co-immunoprecipitation of cohesin component SMC1 (B). (PDF) [file pgen.1004413.s007.pdf]

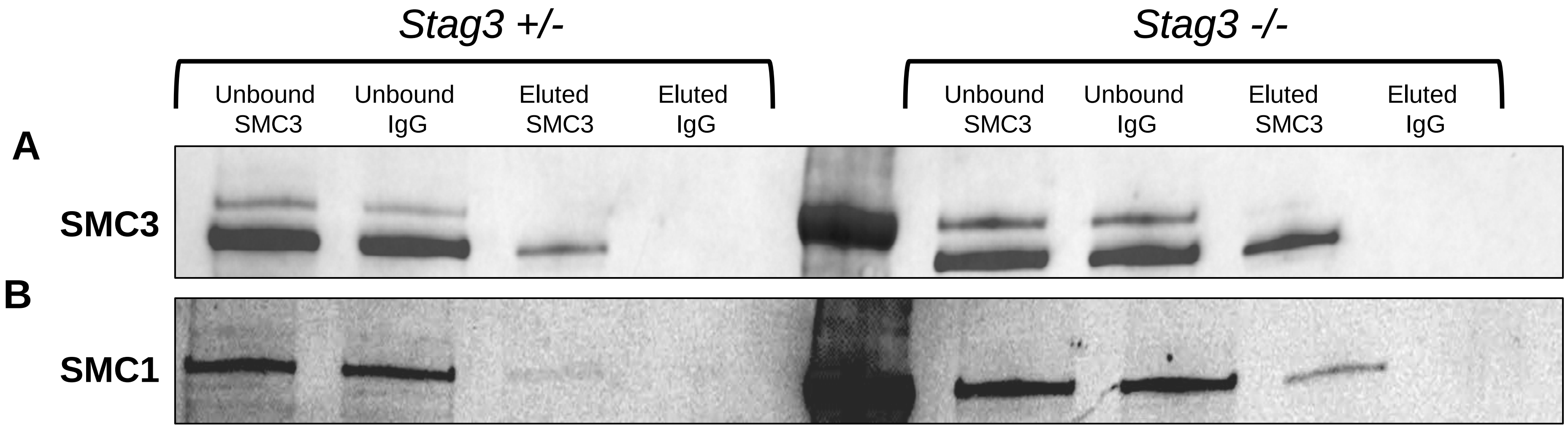
Stag3 +/-
Stag3 -/-
Unbound SMC3
Unbound IgG
Eluted SMC3
Eluted IgG
Unbound SMC3
Unbound IgG
Eluted SMC3
Eluted IgG
A
SMC3
B
SMC1
