# Supplementary material for: Meiosis-Specific Cohesin Component, Stag3 Is Essential for Maintaining Centromere Chromatid Cohesion, and Required for DNA Repair and Synapsis between Homologous Chromosomes
Source: PLoS Genet. 2014 Jul 3;10(7):e1004413. doi: 10.1371/journal.pgen.1004413 (PMC4081007; doi:10.1371/journal.pgen.1004413)
Supplement: Table S1 — Fertility tests for Stag3 mutants and controls. Each mouse was mated to wild type mice of corresponding backgrounds, until at least two rounds of pups were produced for the control mice. Stag3 mutant and control males were mated to two wild type females. Stag3 mutant and control females were mated to a single wild type male. (PDF) [file pgen.1004413.s010.pdf]

Supplementary Table S1: Fertility tests for Stag3 mutants and controls

Genotype	Sex	Number tested	Avg. #pups
Stag3 $^{+/OV}$	Male	6	10.4
Stag3 $^{OV/OV}$	Male	6	0
Stag3 $^{+/OV}$	Female	6	11.1
Stag3 $^{OV/OV}$	Female	6	0
Stag3 $^{+/JAX}$	Male	4	7.7
Stag3 $^{JAX/JAX}$	Male	2	0
Stag3 $^{+/JAX}$	Female	4	7.3
Stag3 $^{JAX/JAX}$	Female	2	0
Stag3$^{JAX/OV}$	Male	2	0
Stag3$^{JAX/OV}$	Female	2	0
