# Supplementary material for: Meiosis-Specific Cohesin Component, Stag3 Is Essential for Maintaining Centromere Chromatid Cohesion, and Required for DNA Repair and Synapsis between Homologous Chromosomes
Source: PLoS Genet. 2014 Jul 3;10(7):e1004413. doi: 10.1371/journal.pgen.1004413 (PMC4081007; doi:10.1371/journal.pgen.1004413)
Supplement: Table S2 — Primary antibodies used in this in this study. Animal host, source, catalogue number (where applicable), and dilution for immunofluorescence microscopy or western blot are listed. (PDF) [file pgen.1004413.s011.pdf]

Supplemental Table S2: Primary antibodies used in this study

				Dilution	
**Antibody**	Host	Source	Catalogue number	Immuno-fluorescence	Western blot
**ATR**	Goat	Santa Cruz	sc-1887	1:50	NA
**ATRIP**	Rabbit	Abcam	Ab175221	1:100	NA
**CREST (CEN)**	Human	Antibodies Incorporated	15-235	1:50	NA
**DMC1**	Mouse	Abcam	ab11054	1:100	NA
**HORMAD1**	Rabbit	Atilla Toth	NA	NA	1:2000
**HORMAD2**	Rabbit	Atilla Toth	NA	NA	1:2000
**MLH1**	Mouse	BD Biosciences	554073	1:50	NA
**RAD21**	Rabbit	Abcam	ab154769	1:250	1:2000
**RAD21L**	Rabbit	Alberto Pendas	NA	1:250	1:2000
**RAD51**	Rabbit	Thermo Scientific	PA5-27195	1:100	NA
**REC8**	Rabbit	Karen Schindler		1:500	1:2000
**SMC1**	Rabbit	Abcam	ab21583	1:100	1:2000
**SMC1α**	Rabbit	Abcam	ab133643	NA	1:2000
**SMC1α**	Rabbit	Rolf Jessberger	NA	1:100	1:1000
**SMC1β**	Rabbit	Rolf Jessberger	NA	1:250	1:3000
**SMC3**	Rabbit	Abcam	ab9263	1:250	1:5000
**SMC6**	Rabbit	Abcam	ab18039	1:250	NA
**STAG1**	Goat	Abcam	Ab4455	NA	1:5000
**STAG2**	Goat	Santa Cruz	sc-54512	NA	1:100
**STAG3**	Goat	Santa Cruz	sc-20341	NA	1:100
**SYCP1**	Rabbit	Novus Biologicals	NB300-229	1:500	1:10,000
**SYCP3**	Rat	Mary Ann Handel	NA	1:500	NA
**SYCP3**	Rabbit	Novus Biologicals	NB300-231	1:1000	NA
**SYCP3**	Mouse	Santa Cruz	sc-74569	1:50	NA
**TEX12**	Guinea pig	Christer Höög	NA	1:200	1:2000
**TRF1**	Mouse	Abcam	ab10579	1:100	NA
**TUBA**	Rabbit	Sigma	T9026	NA	1:10,000
**γH2AX**	Mouse	Millipore	05-636	1:500	NA
